# Supplementary material for: Disease progression in osteosarcoma: a multistate model for the EURAMOS-1 (European and American Osteosarcoma Study) randomised clinical trial
Source: BMJ Open. 2022 Mar 4;12(3):e053083. doi: 10.1136/bmjopen-2021-053083 (PMC8900028; doi:10.1136/bmjopen-2021-053083)
Supplement: Supplementary data [file bmjopen-2021-053083supp001.pdf]

Appendix A: Defining states and transitions for the EURAMOS-1 osteosarcoma multi-state model

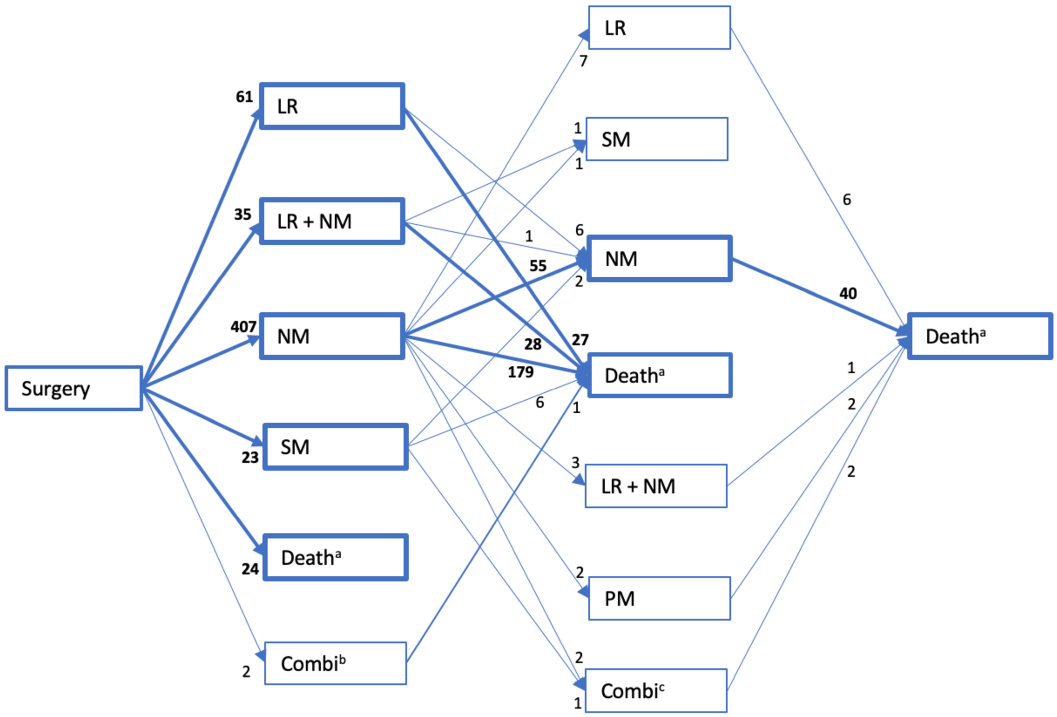

Supplementary Fig. 1 Disease progression of osteosarcoma: all recorded transitions. Eleven possible states and 27 transitions are defined. For each transition, the number of patients progressing from one state to another is shown. A total of 1631 patients are present in the starting stage, Surgery. a) Death is defined as death due to osteosarcoma. Death takes precedence over any other events registered at the same time (e.g. a patient with the combination of NM + Death is considered part of the transition from Surgery to Death, but not the transition from Surgery to NM); b) Combination event: 3 patients with NM+PM; c) Combination event: 1 patient with LR+SM, 1 patient with NM+SM.

Supplementary Fig. 1 gives an overview of all events and transitions recorded in the EURAMOS-1 osteosarcoma data. All patients were considered from their time of surgery, after which a maximum of two consecutive intermediate events was recorded. Our dataset includes a total of 1631 patients who had their primary tumour surgically resected. Of these patients, 552 experienced at least one event, which may be local recurrence (LR, 61), a new metastatic disease (NM, 407), the combination of a local recurrence and a new metastatic disease (LR+NM, 35), a secondary malignancy (SM, 23), a combination of any of the previous events recorded at the same time point (Combi, 2), or death. For example, 24 patients died after surgery without experiencing any of the previously listed events. Another 63 patients experienced a local recurrence, of which 27 died (i.e. transitioned to the state of death), while 6 experienced an NM, and a single patient experienced a second LR.

In our multi-state model, we have only included transitions with sufficient events. The relevant transitions and corresponding states have been marked in bold. Fig. 2 in the main text is a simplified version of the diagram in Supplementary Fig. 1, showing only the states and transitions that are included in our multi-state model. Particular attention must be paid to the definition of these transitions. Consider, for example, the transition from surgery to death, for which we have 25 patients. This particular transition is defined as death without experiencing any of the intermediate events (NM, LR, LR+NM, SM) that are explicitly included in the model. This is not the same as the transition from surgery to death, as defined in Supplementary Fig. 1, where we have 24 patients who die without experiencing any of the recorded intermediate events. For clarity, we have included a diagram of all patients included in the transition from surgery to death, as defined in our multistate model. In Supplementary Fig. 2 we see that there are 24 patients who transition directly from surgery to death, with two patients experiencing a combination event, one of which transitions to death. As the combination event is not included as a state in our model, this patient is counted amongst the surgery-death transition patients, bringing the total to 25 patients.

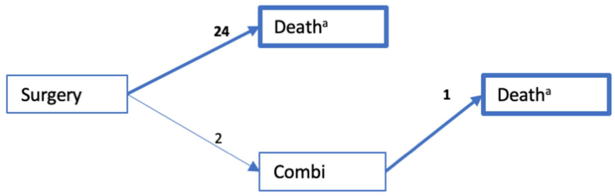

Supplementary Fig. 2 Disease progression of osteosarcoma: the transition from surgery to death, as defined in the multi-state model (Figure 2 in the main text). A total of 25 patients make up the transition from surgery to death in the fitted multi-state model. Of these, 24 progressed to death directly, without any intermediate event, whereas 1 experienced a combination event (NM+PM, not included as a state in the model) prior to death. a) Death is defined as death due to osteosarcoma, and takes precedence over any other events registered at the same time.

Equivalently, we can consider the transition from LR to death, for which we have 30 patients in our multi-state model. In Supplementary Fig. 3 we see that this number is composed of 27 patients who transition directly from LR to death, and 3 patients who experience an NM prior to death. As the latter transition (from LR to NM) is not included in the model, these 3 patients are included in the group of LR-Death transition patients.

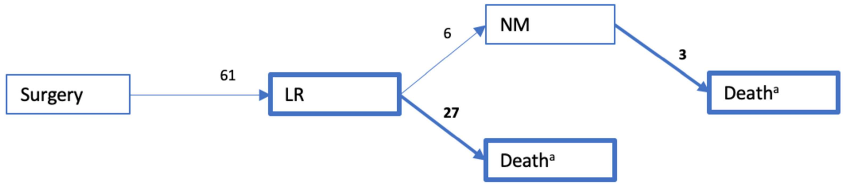

BMJ Publishing Group Limited (BMJ) disclaims all liability and responsibility arising from any reliance placed on this supplemental material which has been supplied by the author(s)

Supplementary Fig. 3 Disease progression of osteosarcoma: the transition from LR to death, as defined in the multi-state model (Figure 2 in the main text). A total of 30 patients make the transition from LR to death in the fitted multi-state model. Of these, 27 progressed to death directly, without any intermediate event, whereas 3 experienced a NM after LR – a transition not included in the multi-state model. a) Death is defined as death due to osteosarcoma, and takes precedence over any other events registered at the same time.

Supplementary Table 1. Event counts per predictor category. Shown are the number of patients per predictor category for each transition. Numbers were computed post multiple imputation, with the final counts averaged across the 10 imputed datasets.

	Surgery ➔ Local recurrence	Surgery ➔ New metastatic disease	Surgery ➔ New metastatic disease + Local recurrence	Surgery ➔ Secondary malignancy	New metastatic disease ➔ New metastatic disease 2	Surgery ➔ Death	Local recurrence ➔ Death	New metastatic disease ➔ Death	New metastatic disease + Local recurrence ➔death	New metastatic disease 2 ➔ Death
**Predictor**	**Event count**									
Age										
12-18	36	228	20	15	33	16	17	111	17	18
<12	**10**	76	**7**	5	11	4	4	30	7	9
>18	15	103	8	3	11	5	9	47	5	6
Histological response										
Good (<10% tumour)	24	162	8	10	22	11	9	66	6	13
Poor (≥10% tumour)	37	245	27	13	33	14	21	122	23	20
Excision										
Wide/radical	49	338	25	19	45	20	23	156	20	27
Marginal	9	61	9	4	7	5	4	28	8	3
Intralesional	3	8	**1**	**0**	3	**0**	3	4	**1**	3
Volume										
<200	41	246	22	19	39	18	18	107	19	20
≥200	20	161	13	4	16	7	12	81	10	13
Sex										
Female	27	161	12	12	21	11	10	63	10	12
Male	34	246	23	11	34	14	20	125	19	21
Location										
Other	38	328	30	20	49	16	13	146	24	28
Proximal	8	67	5	3	6	5	4	34	5	5
Axial	15	12	**0**	**0**	**0**	4	13	8	**0**	**0**
