# Supplementary material for: Disease progression in osteosarcoma: a multistate model for the EURAMOS-1 (European and American Osteosarcoma Study) randomised clinical trial
Source: BMJ Open. 2022 Mar 4;12(3):e053083. doi: 10.1136/bmjopen-2021-053083 (PMC8900028; doi:10.1136/bmjopen-2021-053083)
Supplement: Author's manuscript [file bmjopen-2021-053083.draft_revisions.pdf]

BMJ Open is committed to open peer review. As part of this commitment we make the peer review history of every article we publish publicly available.

When an article is published we post the peer reviewers' comments and the authors' responses online. We also post the versions of the paper that were used during peer review. These are the versions that the peer review comments apply to.

The versions of the paper that follow are the versions that were submitted during the peer review process. They are not the versions of record or the final published versions. They should not be cited or distributed as the published version of this manuscript.

BMJ Open is an open access journal and the full, final, typeset and author-corrected version of record of the manuscript is available on our site with no access controls, subscription charges or pay-per-view fees (http://bmjopen.bmj.com).

If you have any questions on BMJ Open's open peer review process please email info.bmjopen@bmj.com

Disease progression in osteosarcoma: a multi-state model for the EURAMOS-1 randomised clinical trial

Journal:	*BMJ Open*
Manuscript ID	bmjopen-2021-053083
Article Type:	Original research
Date Submitted by the Author:	06-May-2021
Complete List of Authors:	Hazewinkel, Audinga-Dea; Leiden University; University of Bristol, Population Health Sciences<br>Lancia, Carlo; Leiden University<br>Anninga, Jakob; Princess Máxima Centre, Department of Solid Tumours<br>van de Sande, Michiel; Leiden University Medical Center, Department of Orthopaedics<br>Whelan, Jeremy; University College London Hospitals NHS Foundation Trust<br>Gelderblom, Hans; Leiden University Medical Center, Department of Medical Oncology<br>Fiocco, Marta; Leiden University, Mathematical Institute; Leiden University Medical Center, Medical Statistics Department of Biomedical Data Science
Keywords:	Paediatric oncology < ONCOLOGY, Clinical trials < THERAPEUTICS, Sarcoma < ONCOLOGY, Bone diseases < ORTHOPAEDIC & TRAUMA SURGERY

SCHOLARONE™
Manuscripts

BMJ

I, the Submitting Author has the right to grant and does grant on behalf of all authors of the Work (as defined in the below author licence), an exclusive licence and/or a non-exclusive licence for contributions from authors who are: i) UK Crown employees; ii) where BMJ has agreed a CC-BY licence shall apply, and/or iii) in accordance with the terms applicable for US Federal Government officers or employees acting as part of their official duties; on a worldwide, perpetual, irrevocable, royalty-free basis to BMJ Publishing Group Ltd ("BMJ") its licensees and where the relevant Journal is co-owned by BMJ to the co-owners of the Journal, to publish the Work in this journal and any other BMJ products and to exploit all rights, as set out in our licence.

The Submitting Author accepts and understands that any supply made under these terms is made by BMJ to the Submitting Author unless you are acting as an employee on behalf of your employer or a postgraduate student of an affiliated institution which is paying any applicable article publishing charge ("APC") for Open Access articles. Where the Submitting Author wishes to make the Work available on an Open Access basis (and intends to pay the relevant APC), the terms of reuse of such Open Access shall be governed by a Creative Commons licence – details of these licences and which Creative Commons licence will apply to this Work are set out in our licence referred to above.

Other than as permitted in any relevant BMJ Author's Self Archiving Policies, I confirm this Work has not been accepted for publication elsewhere, is not being considered for publication elsewhere and does not duplicate material already published. I confirm all authors consent to publication of this Work and authorise the granting of this licence.

For peer review only

Disease progression in osteosarcoma: a multi-state model for the EURAMOS-1 randomised clinical trial

For peer review only

Author list:

Audinga-Dea Hazewinkel[1,2,3], Carlo Lancia[1], Jakob Anninga[6], Michiel van de Sande[4], Jeremy Whelan[8], Hans Gelderblom[5], Marta Fiocco[1,6,7]

Author affiliations:

1) Mathematical Institute, Leiden University, The Netherlands
2) Population Health Sciences, Bristol Medical School, University of Bristol, United Kingdom
3) MRC Integrative Epidemiology Unit, Bristol Medical School, University of Bristol, United Kingdom
4) Leiden University Medical Center, Department of Orthopaedics, The Netherlands
5) Leiden University Medical Center, Department of Medical Oncology, The Netherlands
6) Princess Maxima Center for Pediatric Oncology, Dept Solid Tumors. Heidelberglaan 25, Utrecht, the Netherlands
7) Leiden University Medical Center, Medical Statistics, Department of Biomedical Data Science, The Netherlands
8) Cancer Institute, Faculty of Medical Sciences, Universtiy College London, United Kingdom

For peer review only - http://bmjopen.bmj.com/site/about/guidelines.xhtml

Corresponding author:

Audinga-Dea Hazewinkel

Oakfield House, Oakfield Grove, University of Bristol, Bristol, England, BS8 2BN,

a.d.hazewinkel@bristol.ac.uk

Running head

A multi-state model for the EURAMOS-1 osteosarcoma clinical trial

For peer review only

Abstract

Objectives: Investigating the effect of prognostic factors in a multi-state framework on survival in a large population of osteosarcoma patients. Of interest is how prognostic factors affect different disease progression stages after surgery, with stages of local recurrence (LR), new metastatic disease (NM), local recurrence + new metastatic disease, secondary malignancy, a second NM, and death.

Design: An open-label, international, phase 3 randomised controlled trial

Setting: 325 sites in 17 countries

Participants: The subset of 1631 metastases-free patients from 1965 patients with high-grade resectable osteosarcoma, from the EURAMOS-I clinical trial.

Main outcome measures: The effect of prognostic factors on different disease stages; predictions of disease progression on an individual patient basis, estimating the probability of occupying a given disease stage according to patient-specific characteristics and history of intermediate events.

Results: Of 1631 patients, 526 experienced an intermediate event, and 305 died by the end of follow-up. An axial tumour site substantially increased the risk of LR after surgery (HR=10.84, 95% CI: 8.46 to 13.86) and death after LR (HR=11.54, 95% CI: 6.11 to 21.8). A poor histological increased the risk of NM (HR=5.81, 95% CI: 5.31 to 6.36), which sharply declined after 3 years since surgery. Young patients (<12 years) had a lower intermediate event risk (e.g., LR: HR=0.66, 95% CI: 0.51 to 0.86), when compared to

For peer review only - http://bmjopen.bmj.com/site/about/guidelines.xhtml

adolescents (12-18 years), but had an increased risk of subsequent death, while patients aged >18 had a decreased risk of death after event (e.g., for death after LR: HR=2.40, 95% CI: 1.52 to 3.90; HR=0.35, 95% CI: 0.21 to 0.56, respectively).

Conclusions: Our findings suggest that patients with axial tumours should be monitored for local recurrence and patients with poor histological response for new metastatic disease, and that young patients (<12) with a local recurrence may benefit from a more intensive treatment.

Trial registration: NCT00143030

Keywords

Osteosarcoma, randomized clinical trial, multi-state, survival, local recurrence, new metastatic disease

Article Summary

Strengths and limitations

1. Osteosarcoma treatment can improve by considering patient-group specific prognosis
2. We estimated a multi-state model for the European and American Osteosarcoma Study-1
3. A multi-state model takes into account patient history and estimates the effect of prognostic factors on the hazard of transitioning to and from specific intermediate events
4. We investigated the effect of prognostic factors on different osteosarcoma disease stages and estimated, for individual patients, the probability of occupying a given stage according to patient-specific characteristics and event history
5. Estimation was limited to transitions with sufficient events per predictor category

For peer review only - http://bmjopen.bmj.com/site/about/guidelines.xhtml

Word count (manuscript, excluding tables, references, and figure headings): 2619 words

For peer review only

For peer review only - http://bmjopen.bmj.com/site/about/guidelines.xhtml

Manuscript

Introduction

Osteosarcoma is the most common primary bone sarcoma, with a primary peak incidence in adolescents and young adults and a second peak in patients of 50 years and older, often due to underlying conditions.[1] Current management strategies include neoadjuvant chemotherapy and surgical removal of the primary tumour and, if resectable, all metastatic disease[2].

The EURAMOS-1 study (NCT00143030) was headed by the EURAMOS (European and American Osteosarcoma Studies) collaboration and recruited a total of 2260 patients from 2005 to 2011.[3] The relationship between various predictors and event-free and overall survival has been investigated previously[4,5,6]. Such analyses, however, only consider one (composite) outcome at the same time, and cannot take into account disease evolution, and how patient history affects the final prognosis.

Here, we reanalyse the EURAMOS-1 data using a multi-state model[7]. A conventional Cox proportional hazards regression defines a single starting state (e.g., study entry) and final state (e.g., death). A multi-state model extends this by introducing intermediate states a patient may transition to (e.g., the development of local recurrence, metastatic disease). The aim of this study is to investigate the effect of prognostic factors on different disease stages, and predict disease progression on an individual patient basis, estimating the probability of occupying a given stage according to patient-specific characteristics and history of intermediate events.

Patients and Methods

Patients

The EURAMOS-1 trial data, per November 2014, contain information on 2260 patients, of which 1336 were randomized, and include predictor measurements and records of intermediate events observed during follow-up.[3] The primary analysis found no beneficial effect of experimental treatment in both good and poor responding groups.[4,5] Here, we consider randomized as well as non-randomized patients,

For peer review only - http://bmjopen.bmj.com/site/about/guidelines.xhtml

and investigate, of all registered patients, the subset with resectable disease and no metastases at baseline. Follow-up is defined from surgery (resection of the primary tumour), performed post neoadjuvant chemotherapy. A total of 1631 patients were considered to be eligible for analysis (Figure 1). The maximum follow-up time was 9 years, with a median of 5 years. Six variables were selected, which have previously been examined in the context of overall and event-free survival.[6] Table 1 shows the distribution of patients across predictor categories. No major differences were observed between randomized and non-randomized patients.

Table 1. Patient demographics

	Treatment randomisation					
	Randomised		Not randomised		Total	
Predictor	N	%	N	%	N	%
Age						
12-18	627	38	275	17	902	55
<12	276	17	117	7	393	24
>18	219	13	117	7	336	21
Missing	0	0	0	0	0	0
Histological response[a]						
Good	614	38	231	14	845	52
Poor	505	31	240	15	745	46
Missing	3	0	38	2	41	3
Excision						
Wide/radical	923	57	404	25	1327	81
Marginal	149	9	55	3	204	13
Intralesional	15	1	7	0	22	1
Unknown[b]	11	1	5	0	16	1
Missing	24	1	38	2	62	4
Volume[c]						
<200	633	39	303	19	936	57
≥200	265	16	126	8	391	24
Missing	224	14	80	5	304	19
Sex						
Female	470	29	220	13	690	42
Male	652	40	289	18	941	58
Missing	0	0	0	0	0	0
Tumour location[d]						
Other	956	59	413	25	1369	84
Proximal femur/humerus	133	8	74	5	207	13

For peer review only - http://bmjopen.bmj.com/site/about/guidelines.xhtml

Axial	33	2	22	1	55	3
Missing	0	0	0	0	0	0

a) A good and poor histological response is defined by the amount of tumour remaining after resection <10% and ≥10% constitute a good and poor response, respectively.

b) A subset of patients had an excision marked as “unknown”. In the analysis this category is treated as missing data and imputed.

c) Absolute volume is measured in cm X cm X cm X 0.54 (spheric T vol), or 0.785 for cylindric T vol)

d) Tumour location was defined in accordance with the definition used in the Smeland *et al.* (2019) analysis of survival and prognosis in the EURAMOS-1 trial. Information was pooled from study variables ‘site’ (eg. femur, pelvis, spine, etc.) and ‘location’ (e.g. proximal, axial, etc.)

Statistical analysis

Multi-state model

In the EURAMOS data, information on various post-surgical events was recorded, with up to two intermediate events reported per patient. Supplementary Fig. 1 (Appendix A) gives an overview of all events and transitions recorded in the data. We selected all intermediate states and transitions that had sufficient events to ensure reliable estimation. We distinguish the following intermediate events: local recurrence (LR), new metastatic disease (NM), the combination of both (LR+NM) and secondary malignancy (SM). New metastatic disease (NM) includes new pulmonary metastases (60%), bone metastases (7%), metastases at other sites (19%) and any combination of the three (14%).

In our model, surgical resection of the primary tumour was chosen as a starting state, with 1631 patients. Of these, 526 experienced an intermediate event, moving to the states of either LR (n=61), NM (n=407), LR+NM (n=35), or SM (n=23), with 280 patients transitioning to death afterwards. An additional 25 patients died without experiencing an intermediate event. Of the 407 patients experiencing a new metastatic disease, 55 experienced a second metastatic disease (NM2) after remission, with 33 patients dying subsequently.

Figure 2 shows a schematic representation of the fitted multi-state model, which consists of seven states and 10 transitions. Appendix A, with supplementary Fig. 1, 2 and 3, provides additional detail on model choice and the definition of states and transitions.

For peer review only - http://bmjopen.bmj.com/site/about/guidelines.xhtml

Time varying effect

The model was stratified on transition, allowing a separate baseline for each of the seven transitions. Histological response violated the proportional hazards assumption for the transitions from surgery to NM and NM to death, indicating that the hazard did not remain constant over time. To account for the time-varying effect, an interaction term of histological response with the exponent of time was included.

Missing data

Of the 1631 patients, 1264 patients were complete cases, with the greatest missingness observed for absolute tumour volume (19%). To make full use of the available data, missing values were imputed in a 10-fold imputation approach. Pooled estimates of the coefficients and standard deviations were obtained using Rubin's rule.[8] For the predictor surgical excision an 'unknown' excision was reported for 16 patients. A sensitivity analysis was conducted by evaluating estimate consistency across three different models, with unknown excision patients excluded from the data, with unknown excision modelled as a separate predictor category, and with unknown excisions treated as missing data and imputed. In the absence of substantial differences in model estimates the latter option was selected.

The statistical analysis was performed in the R-software environment (R version 3.4.2)[9], with the *mstate*[10] and *Amelia*[11] libraries.

Patient and public involvement

Patients or the public were not involved in the design, conduct, reporting or dissemination plans of our research (a re-analysis of the EURAMOS-1 trial using a multi-state approach).

For peer review only - http://bmjopen.bmj.com/site/about/guidelines.xhtml

For peer review only

Results

Transition-specific hazard ratios

The multi-state model is illustrated in Figure 2, along with the number of events. Hazard ratios along with 95% confidence intervals were estimated with a multivariate Cox proportional hazard regression model, modelling all transitions with sufficient events (Supplementary Table 1, Appendix A). Table 2 and 3 show the estimates for all transitions from surgery to intermediate event, and for all transitions terminating in death, respectively.

An axial tumour site, compared to any other limb site except proximal, was associated with a highly increased risk of LR after surgery (HR=10.84, 95% CI: 8.46-13.86, Table 2), death after LR (HR=11.54, 95% CI: 6.11 -21.78, Table 3), and event-free death, i.e. patients who move directly from surgery to death (HR=6.92, 95% CI: 4.50-10.65, Table 3), and with a moderate increased risk of death after NM (HR=1.34, 95% CI: 1.02-1.76, Table 3). Patients with an axial tumour were comparatively less likely to experience an NM after surgery (HR=0.70, 95% CI: 0.56-0.87, Table 2).

Proximal tumour location was associated with moderately increased risks of LR (HR=1.60, 95% CI: 1.21-2.12), NM (HR=1.41, 95% CI: 1.28-1.56), event-free death (HR=2.30, 95% CI: 1.59-3.34), death after LR (HR=2.72, 95% CI: 1.70-4.35), and death after NM (HR=1.33, 95% CI: 1.15-1.53) .

A poor histological response was associated with an increased risk of NM (HR=5.81, 95% CI: 5.31-6.36), LR+NM (HR=3.82, 95% CI: 2.86-5.10), SM (HR=2.07, 95% CI: 1.48-2.90) and LR (HR=1.76, 95% CI: 1.44-2.13). Poor histological response was also associated with an increased risk of event-free death (HR=1.55, 95% CI: 1.15-2.10), and an increased risk of death in patients who had experienced LR+NM (HR=2.45, 95% CI: 1.56-3.86), only LR (HR=1.75, 95% CI: 1.24-2.47), or NM (HR=1.47, 95% CI: 1.29-1.70).

An intralesional surgical excision, compared to a wide or radical one, was strongly associated with an increased risk of a second NM (HR=4.10, 95% CI: 2.61-6.43), death after experiencing a second NM (HR=5.30, 95% CI: 2.86-9.29), and more modestly with LR after surgery (HR=2.08, 95% CI: 1.32-3.29). Patients with either an intralesional or marginal surgical excision were less likely to progress from LR to death (HR=0.33, 95% CI: 0.16-0.67; HR=0.38, 95% CI: 0.22-0.75, respectively).

Younger patients aged <12, compared to adolescents aged 12-18, had a decreased risk of LR (HR=0.66, 95% CI: 0.51-0.86), NM (HR=0.80, 95% CI: 0.72-0.88), SM (HR=0.61, 95% CI: 0.42-0.89), and event-free

For peer review only - http://bmjopen.bmj.com/site/about/guidelines.xhtml

Table 2. Hazard ratio estimates from multi-state model per transition, for all transitions from surgery to intermediate event

	Surgery ➔ Local recurrence		Surgery ➔ New metastatic disease		Surgery ➔ New metastatic disease + Local recurrence		Surgery ➔ Secondary malignancy		New metastatic disease ➔ New metastatic disease 2	
Predictor	**HR**	**95% CI**	**HR**	**95% CI**	**HR**	**95% CI**	**HR**	**95% CI**	**HR**	**95% CI**
Age										
12-18	1		1		1		1		1	
<12	**0.662**	**0.509 - 0.861**	**0.798**	**0.724 - 0.88**	0.844	0.612 - 1.165	**0.612**	**0.42 - 0.893**	1.024	0.793 - 1.323
>18	0.868	0.688 - 1.094	1.088	0.996 - 1.187	0.933	0.689 - 1.265	**0.575**	**0.363 - 0.91**	**0.672**	**0.519 - 0.869**
Histological response										
Good (<10% tumour)	1		1		1		1		1	
Poor (≥10% tumour)	**1.755**	**1.444 - 2.133**	**5.814**	**5.314 - 6.361**	**3.818**	**2.856 - 5.104**	**2.072**	**1.479 - 2.904**	1.052	0.859 - 1.289
Time effect			**0.986**	**0.985 - 0.987**						
Excision										
Wide/radical	1		1						1	
Marginal	0.779	0.587 - 1.033	1.096	0.985 - 1.219					0.862	0.625 - 1.189
Intralesional	**2.082**	**1.32 - 3.285**	1.212	0.938 - 1.566					**4.095**	**2.609 - 6.428**
Location										

death (HR=0.56, 95% CI: 0.37-0.84). However, younger patients had an increased risk of death after LR (HR=2.40, 95% CI: 1.52-3.90), LR+NM (HR=2.84, 95% CI: 1.83-4.41), and NM2 (HR=2.34, 95% CI: 1.69-3.22). Patients older than 18 years experienced a decreased risk of SM (HR=0.58, 95% CI: 0.36-0.91), NM2 (HR=0.67, 95% CI: 0.52-0.87), death after LR (HR=0.35, 95% CI: 0.21-0.56), death after LR+NM (HR=0.47, 95% CI: 0.31-0.71), and death after NM2 (HR=0.53, 95% CI: 0.34-0.83).

Male sex was associated with an increased risk of LR+NM (HR=1.40, 95% CI: 1.08-1.82), NM2 (HR=1.34, 95% CI: 1.09-1.65), death after NM (HR=1.59, 95% CI: 1.42-1.79), and death after LR+NM (HR=1.82, 95% CI: 1.30-2.55), but a decreased risk of SM (HR=0.72, 95% CI: 0.53-0.98).

A tumour volume of >200 was associated with a modest increase in risk of NM (HR=1.51, 95% CI: 1.40-1.64) and LR+NM (HR=1.50, 95% CI: 1.15-1.95), and a decrease in risk of NM2 (HR=0.76, 95% CI: 0.59-0.98).

For peer review only

For peer review only - http://bmjopen.bmj.com/site/about/guidelines.xhtml

Other	1		1							
Proximal femur/humerus	**1.601**	**1.208 - 2.123**	**1.410**	**1.278 - 1.556**						
Axial	**10.837**	**8.458 - 13.885**	**0.700**	**0.564 - 0.869**						
Sex										
Female	1		1		1		1		1	
Male	0.947	0.783 - 1.145	1.048	0.972 - 1.13	**1.402**	**1.079 - 1.82**	**0.724**	**0.534 - 0.982**	**1.343**	**1.092 - 1.652**
Volume										
<200	1		1		1		1		1	
≥200	0.802	0.642 - 1.001	**1.513**	**1.397 - 1.638**	**1.499**	**1.152 - 1.951**	0.755	0.502 - 1.134	**0.760**	**0.592 - 0.975**

Table 3. Hazard ratio estimates from multi-state model per transition, for all absorbent transitions from intermediate event to death

	Surgery ➔ Death		Local recurrence ➔ Death		New metastatic disease ➔ Death		New metastatic disease + Local recurrence ➔death		New metastatic disease 2 ➔ Death	
Predictor	**HR**	**95% CI**	**HR**	**95% CI**	**HR**	**95% CI**	**HR**	**95% CI**	**HR**	**95% CI**
Age										
12-18	1		1		1		1		1	
<12	**0.555**	**0.368 - 0.836**	**2.401**	**1.517 - 3.799**	0.881	0.757 - 1.024	**2.838**	**1.825 - 4.413**	**2.335**	**1.694 - 3.218**
>18	0.739	0.506 - 1.081	**0.345**	**0.213 - 0.560**	0.892	0.785 - 1.014	**0.473**	**0.314 - 0.711**	**0.529**	**0.338 - 0.829**
Histological response										
Good (<10% tumour)	1		1		1		1		1	
Poor (≥10% tumour)	**1.551**	**1.147 - 2.099**	**1.748**	**1.236 - 2.471**	**1.472**	**1.29 - 1.679**	**2.453**	**1.561 - 3.855**	0.872	0.645 - 1.179
Time effect					**0.979**	**0.967 - 0.992**				
Excision										
Wide/radical			1		1				1	
Marginal			**0.379**	**0.221 - 0.651**	1.089	0.929 - 1.277			0.811	0.442 - 1.490
Intralesional			**0.331**	**0.163 - 0.673**	1.401	0.975 - 2.014			**5.296**	**2.856 - 9.819**
Location										

For peer review only - http://bmjopen.bmj.com/site/about/guidelines.xhtml

Other	1		1		1					
Proximal femur/humerus	**2.299**	**1.585 - 3.335**	**2.717**	**1.696 - 4.354**	**1.326**	**1.151 - 1.527**				
Axial	**6.919**	**4.497 - 10.65**	**11.535**	**6.113 - 21.767**	**1.335**	**1.016 - 1.755**				
Sex										
Female	1		1		1		1		1	
Male	0.986	0.733 - 1.326	1.305	0.923 - 1.844	**1.589**	**1.418 - 1.782**	**1.823**	**1.302 - 2.552**	1.165	0.836 - 1.623
Volume										
<200	1		1		1		1		1	
≥200	1.093	0.772 - 1.548	0.756	0.516 - 1.106	1.03	0.915 - 1.16	1.13	0.804 - 1.589	0.866	0.591 - 1.271

For peer review only

Time-varying effect

Histological response violated the proportional hazards assumption for the transition surgery to NM and NM to death. Therefore, the effect of histological response is modelled as function of time. The HR of transitioning to NM decreased with time, initially slowly, and more quickly around the three-year mark (Figure 3A). For the transition from NM to death, the decrease was less pronounced (Figure 3B). The time-varying hazard ratio can be computed for any given time-point using the formula shown below. For example, the HR for the transition from surgery to NM is given as:

$$HR = constant \times time\text{-}varying\text{-}effect^{exp(t)}.$$

Here, *constant* is the surgery to NM HR of 5.81, the *time-varying effect* is 0.986 (Table 2), and *t* is time in years. At 1 year, the hazard ratio is: $HR = 5.81 \times 0.985^{exp(1)} = 5.76$. At three years, the HR has decreased to $5.81 \times 0.985^{exp(3)} = 4.36$.

State-occupation probability plots

Figure 4 illustrates, for a set of example patients, osteosarcoma disease progression through state occupation probability plots, where the probability of being in a given state is plotted against time, in years since surgery. Whereas hazard ratios only inform on the relative change in risk, state occupation probabilities show the absolute probability of being in a given state (i.e., having experienced an

For peer review only - http://bmjopen.bmj.com/site/about/guidelines.xhtml

intermediate event) over a period of time. Panels 1A to 1C illustrate the effect of age category, with in panel 1A the state occupation probabilities of a patient aged 12-18, with all other characteristics reference categories (good histological response, wide/radical excision, tumour volume <200 cm^3, female, tumour location of category 'other'), and in panels 1B and 1C the state occupation probabilities of patients aged <12 and >=18, respectively. Patients aged 12-18 were least likely to remain event-free in the state of surgery (green) and had a moderately higher probability of death (purple). Patients aged <12 were most likely to remain event-free with a lower probability of an intermediate event or death. Patients aged >=18 were at greater risk of NM (blue) than both other age groups.

When compared to the reference patient (1A), patients with an axial osteosarcoma (2A) were at lower risk of NM, higher risk of death, and a much-increased risk of LR (yellow), and were most likely to experience the latter event 1 to 3 years after surgery. An intralesional surgical excision (2B), in contrast, resulted an in increased risk of LR that remained relatively constant over time. Patients with a poor histological response (2C) were more likely to experience NM, or an SM (red). Panels 3A-3C and 4A-4C show the state occupation probabilities for patients who experienced an NM or LR, respectively, at 1, 2 and 4 years after surgery. The later the event occurred, the lower the probability of the disease progressing to death. For example, for LRs 1 year and 4 years after surgery, the 4-year survival probabilities were approximately 0.65 (4A) and 0.8 (4C), respectively.

Discussion

This study is unique in considering the hazard of transitions to and from different intermediate events, allowing for a better insight into prognosis of specific patient groups compared to overall prognostic factors. Using a metastasis free subset of the EURAMOS-1 data[6], the effect of various prognostic factors on disease course are interpreted in a multi-staged way. The most remarkable adverse risk factor was axial location of the osteosarcoma. A previous analysis of the EURAMOS-1 data found moderate HRs of 1.74 (95% CI: 1.06-2.85) and 1.29 (95% CI: 0.86-1.95) for overall survival (OS) and event-free survival (EFS), respectively.[6] In our results, an axial location was chiefly relevant to the transition from surgery to LR and LR to death, yielding substantially higher transition-specific hazard ratios of 10.84 (95% CI: 8.45-13.86) and 11.54 (95% CI: 6.11-21.77), respectively. In contrast, the occurrence of an NM was less

For peer review only - http://bmjopen.bmj.com/site/about/guidelines.xhtml

common (HR=0.70, 95% CI: 0.56-0.87). Clinically, this may have implications for additional treatment and follow-up for osteosarcomas at this site.

A poor histological response has widely been shown to adversely affect overall survival. [6,12-14] The previous EURAMOS-1 analysis found HRs of 2.14 (95% CI: 1.76-2.58) and 2.45 (95% CI: 1.88-3.20) for EFS and OS, respectively. In our study, poor histological response increased the risk of events NM nearly 6x (HR 5.81, 95% CI: 5.31-6.36) and LR+NM nearly 4x (HR 3.81, 95% CI: 2.86-5.10), was predictive of death chiefly after an immediate event, but only modestly affected the risk of an event-free death. In particular, the risk of NM was substantially increased during the first 2.5 years, but the predictive strength steeply decreased after 3 years since surgery, with the HR nearly halved at 4 years follow-up.

For an intralesional excision, compared to a wide/radical excision, HRs of 2.73 (95% CI: 1.15-6.47) and 1.98 (95% CI: 0.91-4.30) were previously found for OS and EFS, respectively.[6] Our results confirm a higher probability of an LR after intralesional surgery (HR 2.08, 95% CI: 1.32-3.29), with this risk remaining comparatively constant during follow-up. Our study shows that developing recurrent NM is a risk factor of intralesional surgery (HR 4.10, 95% CI: 2.61-6.43), with the risk of a subsequent poor outcome increased more than 5x (HR 5.30, 95% CI: 2.86-9.82), substantially higher than the previously reported OS HR.

The remaining variables in our model had more modest HRs, which varied less across transitions. Previously, male sex was found to have only a modest adverse effect on EFS and OS.[6,15] We observed an increased risk of LR+NM and NM2, and subsequent death, but a decreased risk of SM. For greater tumour volume, an EFS HR of 1.24 (95% CI: 1.00-1.52) was previously found[6]. We observed an increased risk for the events NM and LR+NM, with no significant effect for any transition to death, which is in line with previous results for OS (HR=1.19, 95% CI: 0.92-1.55).[5]

Young age was found to be protective for OS and EFS in the previous EURAMOS-1 analysis[6], and other studies.[15,16] However, in Collins' study[15], the hazard lost its significance for survival, at 2 years post-surgery, and our study did not confirm increased risk of event. We found that both the youngest patients (< 12 yr) and those > 18 were protected from experiencing an event. Given the occurrence of LR, LR+NM or NM2, the probability of poor outcome was highest in the young age group, whereas patients > 18 year had a remarkably better outcome. This may have implications for the treatment of LR

For peer review only - http://bmjopen.bmj.com/site/about/guidelines.xhtml

in younger versus older patients, suggesting that older patients could be treated by local treatment only, whereas for younger patients, a more intensified treatment regimen with additional systemic treatment should be considered.

Conclusion

Using data of more than 1600 patients from the EURAMOS-1 trial, we estimated a multi-state model with intermediate events. This study shows the added value of considering prognostic factors specific to transition and in light of event history. Our findings suggest that young patients with a local recurrence may benefit from a more intensive treatment regimen, and stress the necessity of increased monitoring of patients with axial tumours for local recurrences, and patients with a poor histological response for new metastatic disease, while noting for the latter that predictive power decreases over time. We show that a multi-state model yields additional clinical knowledge for specific osteosarcoma patient groups, when compared to conventional OS and EFS analyses. Previously, multi-state models have been used to model disease progression in soft tissue sarcoma[17,18], Ewing sarcoma[19] and breast cancer[20]. Re-analyzing data from other large randomized studies, using the multi-state approach, may also yield valuable insights for patients with other oncological conditions.

Contributorship statement

Audinga-Dea Hazewinkel: Writing- original draft preparation, Visualization, Formal analysis, Software. *Carlo Lancia:* Data curation, Writing- Review & Editing. *Jakob Anninga*: Writing- Review & Editing. *Michiel van de Sande***:** Writing- Review & Editing. *Jeremy Whelan***:** Investigation. *Hans Gelderblom***:** Conceptualization, Writing- Review & Editing. *Marta Fiocco:* Conceptualization, Supervision, Writing- Review & Editing

Competing interests

The authors declare no conflict of interest.

Funding

For peer review only - http://bmjopen.bmj.com/site/about/guidelines.xhtml

We thank the EURAMOS-1 trial investigators and participants.

This work was supported by the Dutch Foundation KiKa (Stichting Kinderen Kankervrij), grant 163, through the project *Meta-analysis of individual patient data to investigate dose-intensity relation with survival outcome for osteosarcoma patients*. AH was supported by Integrative Epidemiology Unit, which receives funding from the UK Medical Research Council and the University of Bristol (MC_UU_00011/3).

Data sharing statement

The data from the EURAMOS-1 trial are not publicly available

Ethics statement

Permission to recruit patients to the protocol was provided by the appropriate national and local regulatory and local committees. Link anonymized data were used for the purposes of this study, and the use of the data was in accordance with the consent taken.

References

1. Gorlick R, Bielack S, Teot L, et al: Osteosarcoma: Biology, diagnosis, treatment, and remaining challenges, in Pizzo P, Poplack D (eds): *Principles and Practice of Pediatric Oncology* 6: 1015-1044, 2011
2. Isakoff MS, Bielack SS, Meltzer P, et al. Osteosarcoma: Current Treatment and a Collaborative Pathway to Success. *J Clin Oncol* 33(27):3029-3035, 2015
3. Whelan JS, Bielack SS, Marina N, et al: EURAMOS-1, an international randomised study for osteosarcoma: Results from pre-randomisation treatment. *Ann Oncol* 26:407-414, 2015
4. Bielack SS, Smeland S, Whelan JS, et al: Methotrexate, Doxorubicin, and Cisplatin (MAP) Plus Maintenance Pegylated Interferon Alfa-2b Versus MAP Alone in Patients With Resectable High-Grade Osteosarcoma and Good Histologic Response to Preoperative MAP: First Results of the EURAMOS-1 Good Response Randomized Controlled Trial. *Journal of Clinical Oncology* 33: 2279–2287, 2015
5. Marina, NM, Smeland S, Bielack SS, et al: Comparison of MAPIE versus MAP in patients with a poor response to preoperative chemotherapy for newly diagnosed high-grade osteosarcoma (EURAMOS-1): an open-label, international, randomised controlled trial. *The Lancet, Oncology* 17: 1396–1408, 2016
6. Smeland S, Bielack SS, Whelan J, et al. Survival and prognosis with osteosarcoma: outcomes in more than 2000 patients in the EURAMOS-1 (European and American Osteosarcoma Study) cohort. *Eur J Cancer*. 2019;109:36-50.
7. Putter H, Fiocco M, R Geskus. Tutorial in biostatistics: competing risks and multi-state models. Stat Med 26:2389–430, 2007
8. Rubin, DB: Multiple imputation after 18+ years. *Journal of the American statistical association.* 91(434):473-489, 1996.
9. R: A language and environment for statistical computing. R Foundation for Statistical Computing, Vienna,

For peer review only - http://bmjopen.bmj.com/site/about/guidelines.xhtml

Austria. http://www.R-project.org
10. Liesbeth C. de Wreede, Marta Fiocco, Hein Putter. Mstate: An R Package for the Analysis of Competing Risks and multi-state models. *Journal of Statistical Software.* 38(7):1-30, 2011
11. Honaker J, King G, Blackwell M. “Amelia II: A Program for Missing Data.” *Journal of Statistical Software*. 45(7):1–47, 2011
12. Rosen G, Murphy ML, Huvos AG, et al: Chemotherapy, en bloc resection, and prosthetic bone replacement in the treatment of osteogenic sarcoma. *Cancer.* 37:1–11, 1976
13. Bacci G, Picci P, Ruggieri P, et al: Primary chemotherapy and delayed surgery (neoadjuvant chemotherapy) for osteosarcoma of the extremities. *Cancer*. 65:2539–2553, 1990
14. Whelan JS, Jinks RC, McTiernan A, et al: Survival from high-grade localised extremity osteosarcoma: combined results and prognostic factors from three European Osteosarcoma Intergroup randomised controlled trials. *Ann Oncol* 23:1607–1616, 2012
15. Collins M, Wilhelm M, Conyers R, et al: Benefits and adverse events in younger versus older patients receiving neoadjuvant chemotherapy for osteosarcoma: findings from a meta-analysis. *J Clin Oncol* 31:2303-12, 2013.
16. Hagleitner MM, Hoogerbrugge PM, van der Graaf WT, et al: Age as prognostic factor in patients with osteosarcoma. *Bone* 49:1173-7, 2012
17. Willeumier JJ, Rueten-Budde AJ, Jeys LM, et al: Individualised risk assessment for local recurrence and distant metastases in a retrospective transatlantic cohort of 687 patients with high-grade soft tissue sarcomas of the extremities: a multistate model. *BMJ Open*. 2017;7:e012930.
18. Posch F, Leitner L, Bergovec M, et al. Can Multistate Modeling of Local Recurrence, Distant Metastasis, and Death Improve the Prediction of Outcome in Patients With Soft Tissue Sarcomas?. *Clin Orthop Relat Res*. 2017;475(5):1427-1435. doi:10.1007/s11999-017-5232-x
19. Bosma SE, Rueten-Budde AJ, Lancia C, et al. Individual risk evaluation for local recurrence and distant metastasis in Ewing sarcoma: A multistate model: A multistate model for Ewing sarcoma. *Pediatr Blood Cancer*. 2019;66(11):e27943. doi:10.1002/pbc.27943
20. Putter H, van der Hage J, de Bock GH, Elgalta R, van de Velde CJ. Estimation and prediction in a multi-state model for breast cancer. *Biom J*. 2006;48(3):366-380. doi:10.1002/bimj.200510218

Figure legends

Figure 1 Consort diagram of patients included in the analysis. In the boxes we list the six exclusion criteria with the total number of patients per category. Above the arrows, we give the additional number of patients excluded on considering each criterion, in order of appearance. a) to ensure a homogeneous study population, we excluded patients with metastases at admission. Additionally, we excluded patients from whom progression of new metastatic disease was found, but no new metastatic disease was recorded.

Figure 2 Disease progression of osteosarcoma represented in a multi-state model. Seven possible states and 10 transitions are defined. For each transition, the number of patients progressing from one state to another is

For peer review only - http://bmjopen.bmj.com/site/about/guidelines.xhtml

shown. A total of 1631 patients are present in the starting stage, Surgery. After surgery, a patient may experience a local recurrence (LR), a local recurrence + new metastatic disease (LR+NM), a new metastatic disease (NM), a secondary malignancy (SM) or death. After any such intermediate event, a patient may progress to death. NM patients may experience a second new metastatic disease after remission (NM2).

Figure 3 Time-varying hazard for histological response. *A*: hazard for transitioning from surgery to new metastatic disease (NM). *B*: hazard for transitioning from NM to death. *Blue*: poor histological response; *black*: good histological response; *Dashed line*: point-wise confidence interval for hazard ratio of poor histological response.

Figure 4 Stacked state occupation probabilities for patients with different characteristics. Patient characteristics are defined with respect to the reference patient, shown in *1A*: patient with reference characteristics – age 12-18 years, good histological response, wide/radical excision, tumour volume <200 cm^3, female, tumour location of category 'other'. *1B*: patient aged <12 years. *1C*: patient aged >=18 years. *2A:* Patient with axial tumour location. *2B*: Patient with poor histological response. *2C*: patient with intralesional excision. *3A-3C*: Patient with reference characteristics experiencing a new metastatic disease (NM) at one year *(3A)*, at two years (*3B)*, and at four years (*3C*). *4A-4C*: Patient with reference characteristics experiencing local recurrence (LR) at one year *(3A)*, at two years (*3B)*, and at four years (*3C*).

For peer review only

For peer review only - http://bmjopen.bmj.com/site/about/guidelines.xhtml

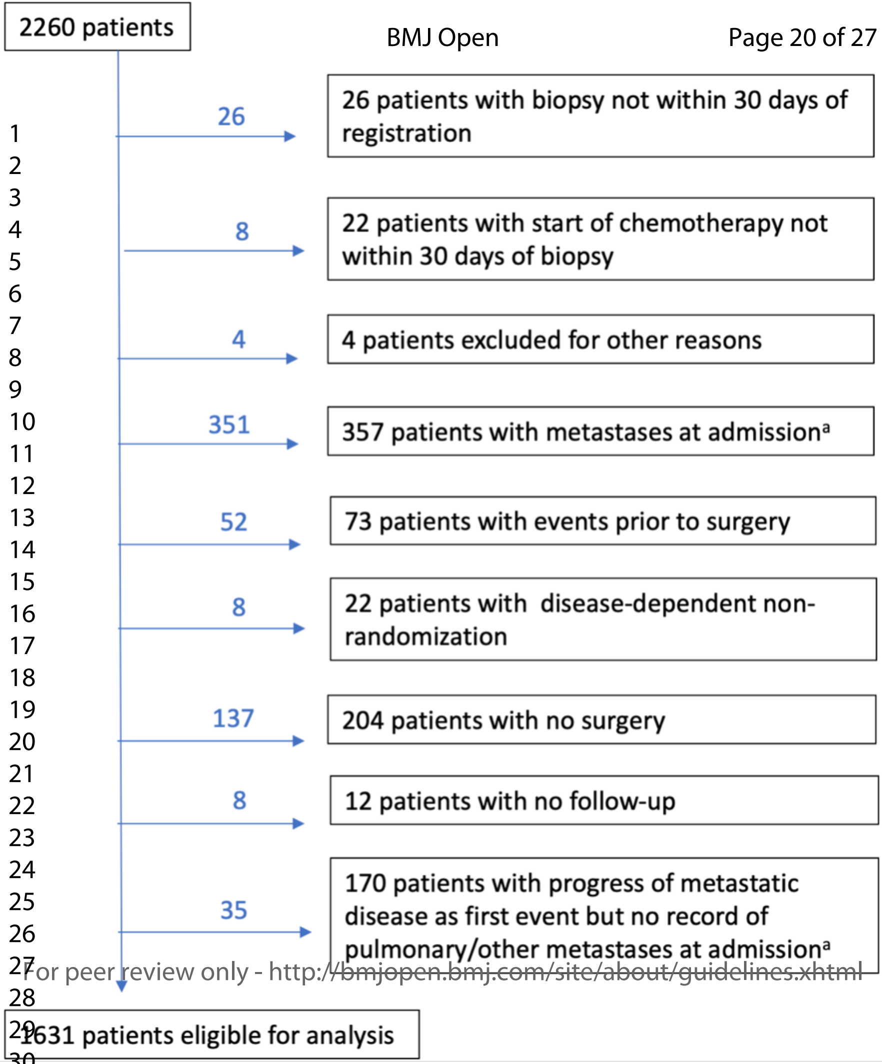
2260 patients
26
26 patients with biopsy not within 30 days of registration
8
22 patients with start of chemotherapy not within 30 days of biopsy
4
4 patients excluded for other reasons
351
357 patients with metastases at admission[a]
52
73 patients with events prior to surgery
8
22 patients with disease-dependent non-randomization
137
204 patients with no surgery
8
12 patients with no follow-up
35
170 patients with progress of metastatic disease as first event but no record of pulmonary/other metastases at admission[a]
1631 patients eligible for analysis

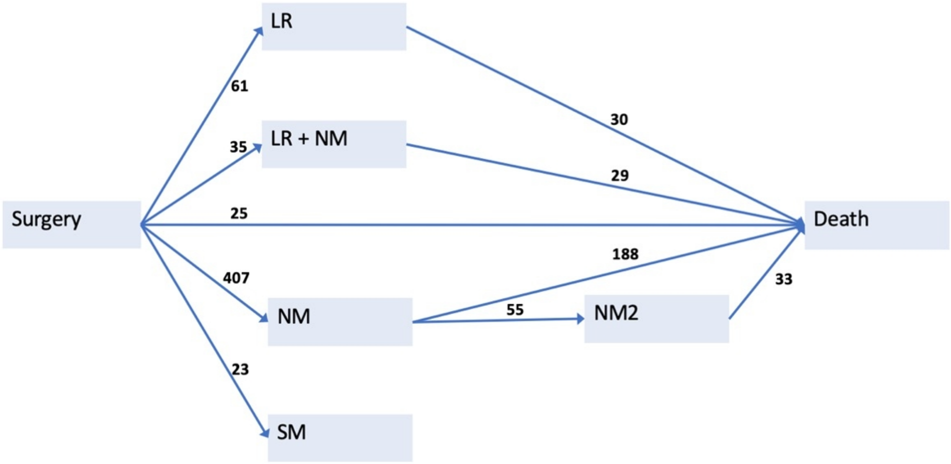

Figure 2 Disease progression of osteosarcoma represented in a multi-state model. Seven possible states and 10 transitions are defined. For each transition, the number of patients progressing from one state to another is shown. A total of 1631 patients are present in the starting stage, Surgery. After surgery, a patient may experience a local recurrence (LR), a local recurrence + new metastatic disease (LR+NM), a new metastatic disease (NM), a secondary malignancy (SM) or death. After any such intermediate event, a patient may progress to death. NM patients may experience a second new metastatic disease after remission (NM2).

141x72mm (220 x 220 DPI)

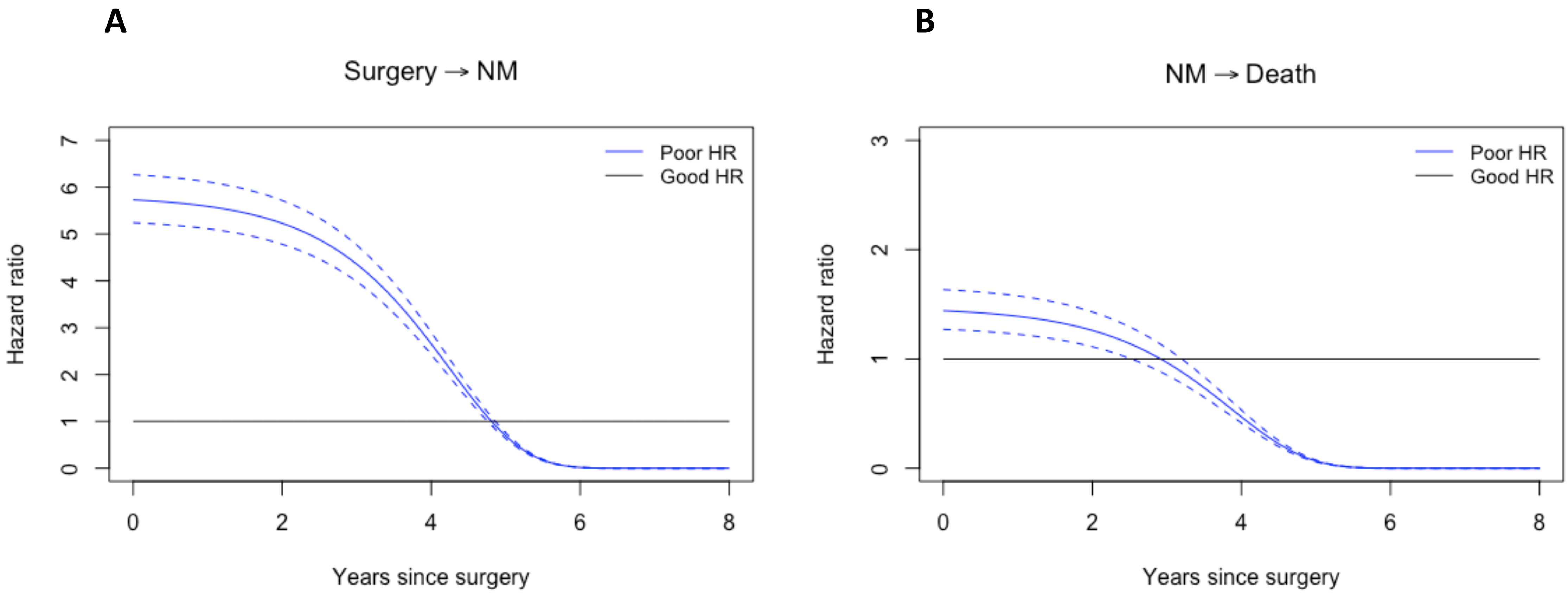
A
Surgery → NM
Hazard ratio
Years since surgery
Poor HR
Good HR
0
1
2
3
4
5
6
7
0
2
4
6
8
B
NM → Death
Hazard ratio
Years since surgery
Poor HR
Good HR
0
1
2
3
0
2
4
6
8

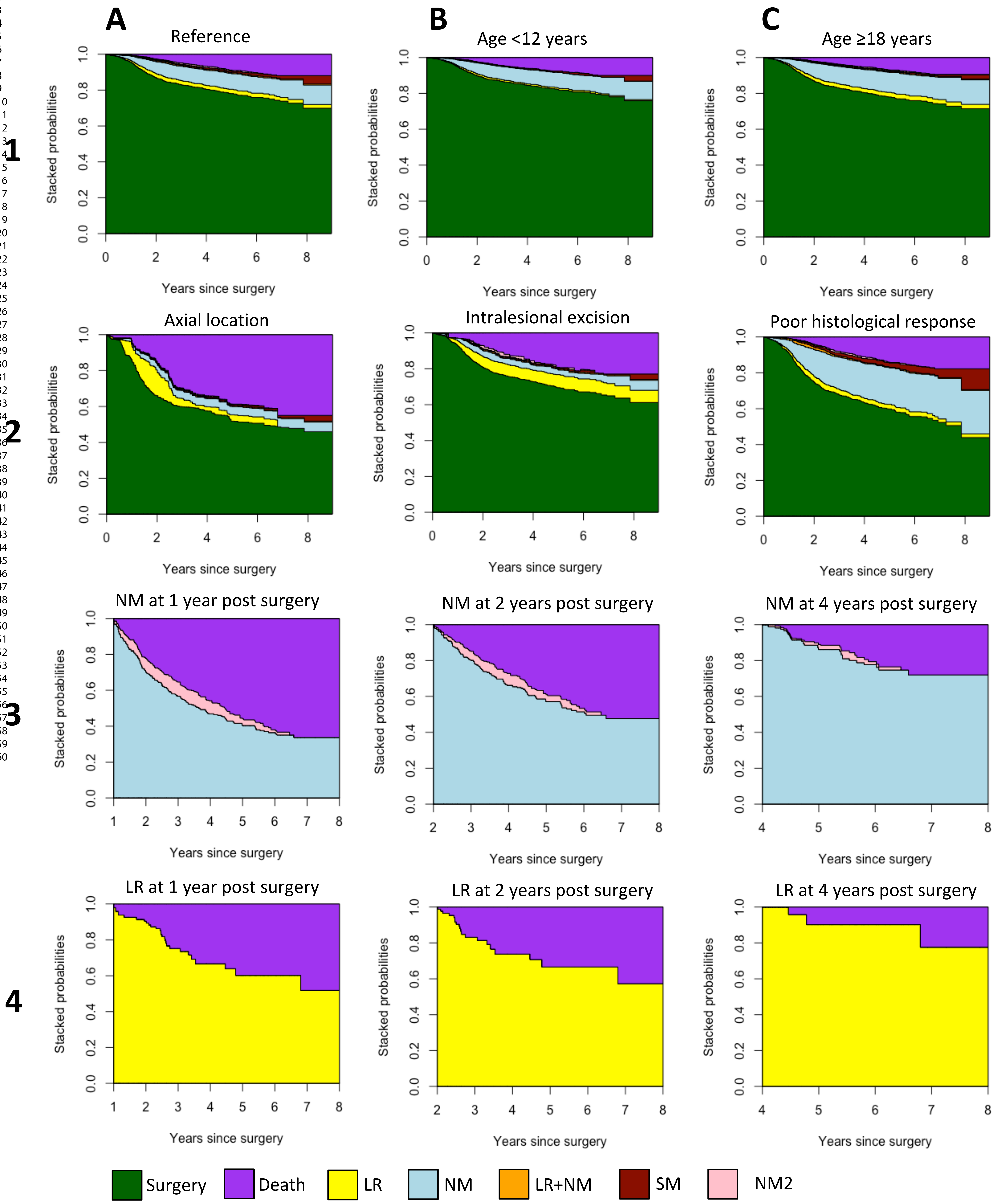
A
B
C
1
2
3
4
Reference
Age <12 years
Age ≥18 years
Axial location
Intralesional excision
Poor histological response
NM at 1 year post surgery
NM at 2 years post surgery
NM at 4 years post surgery
LR at 1 year post surgery
LR at 2 years post surgery
LR at 4 years post surgery
Stacked probabilities
Years since surgery
Surgery
Death
LR
NM
LR+NM
SM
NM2

Appendix A: Defining states and transitions for the EURAMOS-1 osteosarcoma multi-state model

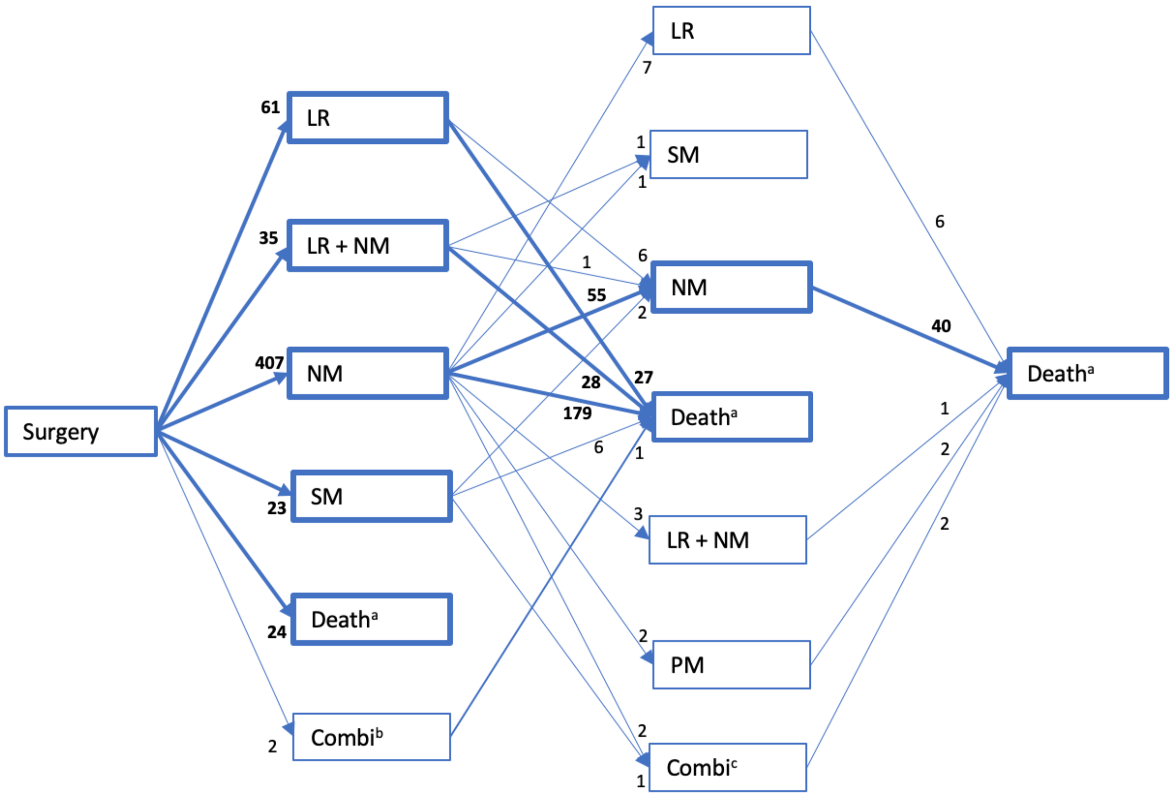

Supplementary Fig. 1 Disease progression of osteosarcoma: all recorded transitions. Eleven possible states and 27 transitions are defined. For each transition, the number of patients progressing from one state to another is shown. A total of 1631 patients are present in the starting stage, Surgery. a) Death is defined as death due to osteosarcoma. Death takes precedence over any other events registered at the same time (e.g. a patient with the combination of NM + Death is considered part of the transition from Surgery to Death, but not the transition from Surgery to NM); b) Combination event: 3 patients with NM+PM; c) Combination event: 1 patient with LR+SM, 1 patient with NM+SM.

Supplementary Fig. 1 gives an overview of all events and transitions recorded in the EURAMOS-1 osteosarcoma data. All patients were considered from their time of surgery, after which a maximum of two consecutive intermediate events was recorded. Our dataset includes a total of 1631 patients who had their primary tumour surgically resected. Of these patients, 552 experienced at least one event, which may be local recurrence (LR, 61), a new metastatic disease (NM, 407), the combination of a local recurrence and a new metastatic disease (LR+NM, 35), a secondary malignancy (SM, 23), a combination of any of the previous events recorded at the same time point (Combi, 2), or death. For example, 24 patients died after surgery without experiencing any of the previously listed events. Another 63 patients experienced a local recurrence, of which 27 died (i.e. transitioned to the state of death), while 6 experienced an NM, and a single patient experienced a second LR.

For peer review only - http://bmjopen.bmj.com/site/about/guidelines.xhtml

In our multi-state model, we have only included transitions with sufficient events. The relevant transitions and corresponding states have been marked in bold. Fig. 2 in the main text is a simplified version of the diagram in Supplementary Fig. 1, showing only the states and transitions that are included in our multi-state model. Particular attention must be paid to the definition of these transitions. Consider, for example, the transition from surgery to death, for which we have 25 patients. This particular transition is defined as death without experiencing any of the intermediate events (NM, LR, LR+NM, SM) that are explicitly included in the model. This is not the same as the transition from surgery to death, as defined in Supplementary Fig. 1, where we have 24 patients who die without experiencing any of the recorded intermediate events. For clarity, we have included a diagram of all patients included in the transition from surgery to death, as defined in our multistate model. In Supplementary Fig. 2 we see that there are 24 patients who transition directly from surgery to death, with two patients experiencing a combination event, one of which transitions to death. As the combination event is not included as a state in our model, this patient is counted amongst the surgery-death transition patients, bringing the total to 25 patients.

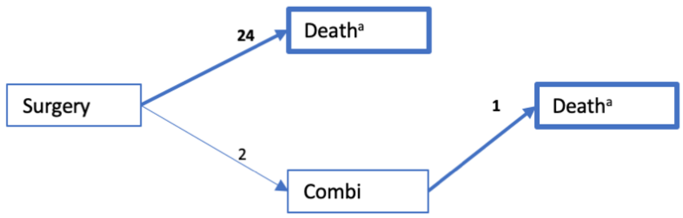

Supplementary Fig. 2 Disease progression of osteosarcoma: the transition from surgery to death, as defined in the multi-state model (Figure 2 in the main text). A total of 25 patients make up the transition from surgery to death in the fitted multi-state model. Of these, 24 progressed to death directly, without any intermediate event, whereas 1 experienced a combination event (NM+PM, not included as a state in the model) prior to death. a) Death is defined as death due to osteosarcoma, and takes precedence over any other events registered at the same time.

Equivalently, we can consider the transition from LR to death, for which we have 30 patients in our multi-state model. In Supplementary Fig. 3 we see that this number is composed of 27 patients who transition directly from LR to death, and 3 patients who experience an NM prior to death. As the latter transition (from LR to NM) is not included in the model, these 3 patients are included in the group of LR-Death transition patients.

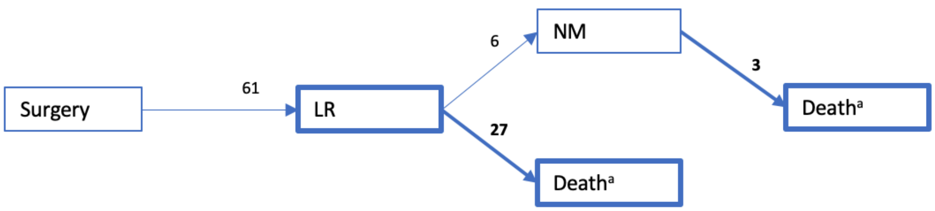

For peer review only - http://bmjopen.bmj.com/site/about/guidelines.xhtml

Supplementary Fig. 3 Disease progression of osteosarcoma: the transition from LR to death, as defined in the multi-state model (Figure 2 in the main text). A total of 30 patients make the transition from LR to death in the fitted multi-state model. Of these, 27 progressed to death directly, without any intermediate event, whereas 3 experienced a NM after LR – a transition not included in the multi-state model. a) Death is defined as death due to osteosarcoma, and takes precedence over any other events registered at the same time.

Supplementary Table 1. Event counts per predictor category. Shown are the number of patients per predictor category for each transition. Numbers were computed post multiple imputation, with the final counts averaged across the 10 imputed datasets.

	Surgery ➔ Local recurrence	Surgery ➔ New metastatic disease	Surgery ➔ New metastatic disease + Local recurrence	Surgery ➔ Secondary malignancy	New metastatic disease ➔ New metastatic disease 2	Surgery ➔ Death	Local recurrence ➔ Death	New metastatic disease ➔ Death	New metastatic disease + Local recurrence ➔death	New metastatic disease 2 ➔ Death
**Predictor**	**Event count**									
Age										
12-18	36	228	20	15	33	16	17	111	17	18
<12	**10**	76	**7**	5	11	4	4	30	7	9
>18	15	103	8	3	11	5	9	47	5	6
Histological response										
Good (<10% tumour)	24	162	8	10	22	11	9	66	6	13
Poor (≥10% tumour)	37	245	27	13	33	14	21	122	23	20
Excision										
Wide/radical	49	338	25	19	45	20	23	156	20	27
Marginal	9	61	9	4	7	5	4	28	8	3
Intralesional	3	8	**1**	**0**	3	**0**	3	4	**1**	3
Volume										
<200	41	246	22	19	39	18	18	107	19	20
≥200	20	161	13	4	16	7	12	81	10	13
Sex										
Female	27	161	12	12	21	11	10	63	10	12
Male	34	246	23	11	34	14	20	125	19	21
Location										
Other	38	328	30	20	49	16	13	146	24	28
Proximal	8	67	5	3	6	5	4	34	5	5
Axial	15	12	**0**	**0**	**0**	4	13	8	**0**	**0**

For peer review only

For peer review only - http://bmjopen.bmj.com/site/about/guidelines.xhtml

CONSORT 2010 checklist of information to include when reporting a randomised trial*

Section/Topic	Item No	Checklist item	Reported on page No
**Title and abstract**			
	1a	Identification as a randomised trial in the title	1
	1b	Structured summary of trial design, methods, results, and conclusions (for specific guidance see CONSORT for abstracts)	3
**Introduction**			
Background and objectives	2a	Scientific background and explanation of rationale	5
	2b	Specific objectives or hypotheses	5
**Methods**			
Trial design	3a	Description of trial design (such as parallel, factorial) including allocation ratio	5
	3b	Important changes to methods after trial commencement (such as eligibility criteria), with reasons	NA
Participants	4a	Eligibility criteria for participants	6
	4b	Settings and locations where the data were collected	3
Interventions	5	The interventions for each group with sufficient details to allow replication, including how and when they were actually administered	NA
Outcomes	6a	Completely defined pre-specified primary and secondary outcome measures, including how and when they were assessed	3
	6b	Any changes to trial outcomes after the trial commenced, with reasons	NA
Sample size	7a	How sample size was determined	NA
	7b	When applicable, explanation of any interim analyses and stopping guidelines	NA
Randomisation:			
Sequence generation	8a	Method used to generate the random allocation sequence	NA
	8b	Type of randomisation; details of any restriction (such as blocking and block size)	NA
Allocation concealment mechanism	9	Mechanism used to implement the random allocation sequence (such as sequentially numbered containers), describing any steps taken to conceal the sequence until interventions were assigned	NA
Implementation	10	Who generated the random allocation sequence, who enrolled participants, and who assigned participants to interventions	NA
Blinding	11a	If done, who was blinded after assignment to interventions (for example, participants, care providers, those	NA

For peer review only - http://bmjopen.bmj.com/site/about/guidelines.xhtml
For peer review only

		assessing outcomes) and how	
	11b	If relevant, description of the similarity of interventions	NA
Statistical methods	12a	Statistical methods used to compare groups for primary and secondary outcomes	7-8
	12b	Methods for additional analyses, such as subgroup analyses and adjusted analyses	7-8
**Results**			
Participant flow (a diagram is strongly recommended)	13a	For each group, the numbers of participants who were randomly assigned, received intended treatment, and were analysed for the primary outcome	5-6
	13b	For each group, losses and exclusions after randomisation, together with reasons	5-6
Recruitment	14a	Dates defining the periods of recruitment and follow-up	5
	14b	Why the trial ended or was stopped	5
Baseline data	15	A table showing baseline demographic and clinical characteristics for each group	6-7
Numbers analysed	16	For each group, number of participants (denominator) included in each analysis and whether the analysis was by original assigned groups	7
Outcomes and estimation	17a	For each primary and secondary outcome, results for each group, and the estimated effect size and its precision (such as 95% confidence interval)	8-13
	17b	For binary outcomes, presentation of both absolute and relative effect sizes is recommended	NA
Ancillary analyses	18	Results of any other analyses performed, including subgroup analyses and adjusted analyses, distinguishing pre-specified from exploratory	NA
Harms	19	All important harms or unintended effects in each group (for specific guidance see CONSORT for harms)	NA
**Discussion**			
Limitations	20	Trial limitations, addressing sources of potential bias, imprecision, and, if relevant, multiplicity of analyses	13-14
Generalisability	21	Generalisability (external validity, applicability) of the trial findings	13-14
Interpretation	22	Interpretation consistent with results, balancing benefits and harms, and considering other relevant evidence	13-14
**Other information**			
Registration	23	Registration number and name of trial registry	3
Protocol	24	Where the full trial protocol can be accessed, if available	5
Funding	25	Sources of funding and other support (such as supply of drugs), role of funders	2

*We strongly recommend reading this statement in conjunction with the CONSORT 2010 Explanation and Elaboration for important clarifications on all the items. If relevant, we also recommend reading CONSORT extensions for cluster randomised trials, non-inferiority and equivalence trials, non-pharmacological treatments, herbal interventions, and pragmatic trials. Additional extensions are forthcoming: for those and for up to date references relevant to this checklist, see www.consort-statement.org.

For peer review only - http://bmjopen.bmj.com/site/about/guidelines.xhtml

For peer review only

Disease progression in osteosarcoma: a multi-state model for the EURAMOS-1 (European and American Osteosarcoma Study) randomised clinical trial

Journal:	*BMJ Open*
Manuscript ID	bmjopen-2021-053083.R1
Article Type:	Original research
Date Submitted by the Author:	02-Dec-2021
Complete List of Authors:	Hazewinkel, Audinga-Dea; Leiden University; University of Bristol, Population Health Sciences<br>Lancia, Carlo; Leiden University<br>Anninga, Jakob; Princess Máxima Centre, Department of Solid Tumours<br>van de Sande, Michiel; Leiden University Medical Center, Department of Orthopaedics<br>Whelan, Jeremy; University College London Hospitals NHS Foundation Trust<br>Gelderblom, Hans; Leiden University Medical Center, Department of Medical Oncology<br>Fiocco, Marta; Leiden University, Mathematical Institute; Leiden University Medical Center, Medical Statistics Department of Biomedical Data Science
**Primary Subject Heading**:	Oncology
Secondary Subject Heading:	Paediatrics, Surgery
Keywords:	Paediatric oncology < ONCOLOGY, Clinical trials < THERAPEUTICS, Sarcoma < ONCOLOGY, Bone diseases < ORTHOPAEDIC & TRAUMA SURGERY

SCHOLARONE™
Manuscripts

I, the Submitting Author has the right to grant and does grant on behalf of all authors of the Work (as defined in the below author licence), an exclusive licence and/or a non-exclusive licence for contributions from authors who are: i) UK Crown employees; ii) where BMJ has agreed a CC-BY licence shall apply, and/or iii) in accordance with the terms applicable for US Federal Government officers or employees acting as part of their official duties; on a worldwide, perpetual, irrevocable, royalty-free basis to BMJ Publishing Group Ltd ("BMJ") its licensees and where the relevant Journal is co-owned by BMJ to the co-owners of the Journal, to publish the Work in this journal and any other BMJ products and to exploit all rights, as set out in our licence.

The Submitting Author accepts and understands that any supply made under these terms is made by BMJ to the Submitting Author unless you are acting as an employee on behalf of your employer or a postgraduate student of an affiliated institution which is paying any applicable article publishing charge ("APC") for Open Access articles. Where the Submitting Author wishes to make the Work available on an Open Access basis (and intends to pay the relevant APC), the terms of reuse of such Open Access shall be governed by a Creative Commons licence – details of these licences and which Creative Commons licence will apply to this Work are set out in our licence referred to above.

Other than as permitted in any relevant BMJ Author's Self Archiving Policies, I confirm this Work has not been accepted for publication elsewhere, is not being considered for publication elsewhere and does not duplicate material already published. I confirm all authors consent to publication of this Work and authorise the granting of this licence.

For peer review only

Disease progression in osteosarcoma: a multi-state model for the EURAMOS-1 (European and American Osteosarcoma Study) randomised clinical trial

Author list:

Audinga-Dea Hazewinkel[1,2,3], Carlo Lancia[1], Jakob Anninga[6], Michiel van de Sande[4], Jeremy Whelan[8], Hans Gelderblom[5], Marta Fiocco[1,6,7]

Author affiliations:

1) Mathematical Institute, Leiden University, The Netherlands
2) Population Health Sciences, Bristol Medical School, University of Bristol, United Kingdom
3) MRC Integrative Epidemiology Unit, Bristol Medical School, University of Bristol, United Kingdom
4) Leiden University Medical Center, Department of Orthopaedics, The Netherlands
5) Leiden University Medical Center, Department of Medical Oncology, The Netherlands
6) Princess Maxima Center for Pediatric Oncology, Dept Solid Tumors. Heidelberglaan 25, Utrecht, the Netherlands
7) Leiden University Medical Center, Medical Statistics, Department of Biomedical Data Science, The Netherlands

For peer review only - http://bmjopen.bmj.com/site/about/guidelines.xhtml

8) Cancer Institute, Faculty of Medical Sciences, Universtiy College London, United Kingdom

For peer review only

Corresponding author:

Audinga-Dea Hazewinkel

Oakfield House, Oakfield Grove, University of Bristol, Bristol, England, BS8 2BN,

a.d.hazewinkel@bristol.ac.uk

Running head

A multi-state model for the EURAMOS-1 osteosarcoma clinical trial

For peer review only

Abstract

Objectives: Investigating the effect of prognostic factors in a multi-state framework on survival in a large population of osteosarcoma patients. Of interest is how prognostic factors affect different disease stages after surgery, with stages of local recurrence (LR), new metastatic disease (NM), local recurrence + new metastatic disease, secondary malignancy, a second NM, and death.

Design: An open-label, international, phase 3 randomised controlled trial

Setting: 325 sites in 17 countries

Participants: The subset of 1631 metastases-free patients from 1965 patients with high-grade resectable osteosarcoma, from the European and American Osteosarcoma Study (EURAMOS-I).

Main outcome measures: The effect of prognostic factors on different disease stages, expressed as hazard ratios (HR); predictions of disease progression on an individual patient basis, according to patient-specific characteristics and history of intermediate events.

Results: Of 1631 patients, 526 experienced an intermediate event, and 305 died by the end of follow-up. An axial tumour site substantially increased the risk of LR after surgery (HR=10.84, 95% CI: 8.46 to 13.86) and death after LR (HR=11.54, 95% CI: 6.11 to 21.8). A poor histological increased the risk of NM (HR=5.81, 95% CI: 5.31 to 6.36), which sharply declined after 3 years since surgery. Young patients (<12 years) had a lower intermediate event risk (e.g., LR: HR=0.66, 95% CI: 0.51 to 0.86), when compared to

For peer review only - http://bmjopen.bmj.com/site/about/guidelines.xhtml

adolescents (12-18 years), but had an increased risk of subsequent death, while patients aged >18 had a decreased risk of death after event (e.g., for death after LR: HR=2.40, 95% CI: 1.52 to 3.90; HR=0.35, 95% CI: 0.21 to 0.56, respectively).

Conclusions: Our findings suggest that patients with axial tumours should be monitored for local recurrence and patients with poor histological response for new metastatic disease, and that for young patients (<12) with a local recurrence additional treatment options should be investigated.

Trial registration: NCT00134030

Keywords

Osteosarcoma, randomised clinical trial, multi-state, survival, local recurrence, new metastatic disease

Article Summary

Strengths and limitations

1. A multi-state model takes into account patient history and estimates the effect of prognostic factors on the hazard of transitioning to and from specific events over the course of disease progression
2. A multi-state model can be used to quantify the risk of experiencing specific disease stages, conditional on prior events and patient characteristics
3. A multi-state model can be used to calculate individual, patient-specific probabilities of occupying a given disease stage
4. Multi-state model estimation is limited to transitions with sufficient events per predictor category

For peer review only

Word count (manuscript, excluding tables, references, and figure headings): 2753 words

For peer review only

For peer review only - http://bmjopen.bmj.com/site/about/guidelines.xhtml

Manuscript

Introduction

Osteosarcoma is the most common primary bone sarcoma, with a primary peak incidence in adolescents and young adults and a second peak in patients of 50 years and older, often due to underlying conditions.[1] Current management strategies include neoadjuvant chemotherapy and surgical removal of the primary tumour and, if resectable, all metastatic disease[2].

The EURAMOS-1 study (NCT00134030) was headed by the EURAMOS (European and American Osteosarcoma Studies) collaboration and recruited a total of 2260 patients from 2005 to 2011.[3] The relationship between various predictors and event-free and overall survival has been investigated previously[4,5,6]. Such analyses, however, only consider one (composite) outcome at the same time, and cannot take into account disease evolution, and how patient history affects the final prognosis.

Here, we reanalyse the EURAMOS-1 data using a multi-state model[7]. A conventional Cox proportional hazards regression defines a single starting state (e.g., study entry) and final state (e.g., death). A multi-state model extends this by introducing intermediate states a patient may transition to (e.g., the development of local recurrence, metastatic disease). The aim of this study is to investigate the effect of prognostic factors on different disease stages, and predict disease progression on an individual patient basis, estimating the probability of occupying a given stage according to patient-specific characteristics and history of intermediate events.

Patients and Methods

Patients

The EURAMOS-1 trial data, per November 2014, contain information on 2260 patients, and include predictor measurements and records of intermediate events observed during follow-up.[3] Resection of the primary tumour was performed post neoadjuvant chemotherapy. Following surgery, 1136 of 2260 patients were randomised to treatment, subject to the histological response as assessed in the resected

For peer review only - http://bmjopen.bmj.com/site/about/guidelines.xhtml

For peer review only

specimen. Patients with a poor response (≥ 10% viable tumour) were allocated MAP or MAP with ifosfamide and etoposide, while patients with a good response (< 10%) received MAP or MAP followed by pegylated interferon. The primary analysis found no beneficial effect of experimental treatment in either group.[4,5] As such, we included both randomised and non-randomised patients in our analysis. To ensure valid inference, we selected a homogeneous subset of the data, excluding patients with a non-resectable primary tumour and patients with clinically detectable metastatic disease prior to surgery, as the latter comprise a biologically distinct population with a much poorer prognosis. A total of 1631 patients were considered to be eligible for analysis (Figure 1). Follow-up is defined from surgery, with a maximum follow-up time of 9 years and a median of 5 years. Six variables were selected, which have previously been examined in the context of overall and event-free survival.[6] Table 1 shows the distribution of patients across predictor categories. No major differences were observed between randomised and non-randomised patients.

Table 1. Patient demographics and disease characteristics

	Treatment randomisation					
	Randomised		Not randomised		Total	
Predictor	N	%	N	%	N	%
Age						
12-18	627	38	275	17	902	55
<12	276	17	117	7	393	24
>18	219	13	117	7	336	21
Missing	0	0	0	0	0	0
Histological response[a]						
Good	614	38	231	14	845	52
Poor	505	31	240	15	745	46
Missing	3	0	38	2	41	3
Excision						
Wide/radical	923	57	404	25	1327	81
Marginal	149	9	55	3	204	13
Intralesional	15	1	7	0	22	1
Unknown[b]	11	1	5	0	16	1
Missing	24	1	38	2	62	4
Volume[c]						
<200	633	39	303	19	936	57
≥200	265	16	126	8	391	24
Missing	224	14	80	5	304	19

For peer review only - http://bmjopen.bmj.com/site/about/guidelines.xhtml

Sex						
Female	470	29	220	13	690	42
Male	652	40	289	18	941	58
Missing	0	0	0	0	0	0
Tumour location[d]						
Other	956	59	413	25	1369	84
Proximal femur/humerus	133	8	74	5	207	13
Axial	33	2	22	1	55	3
Missing	0	0	0	0	0	0

a) A good and poor histological response is defined by the amount of tumour remaining after resection <10% and ≥10% constitute a good and poor response, respectively.

b) A subset of patients had an excision marked as "unknown". In the analysis this category is treated as missing data and imputed.

c) Absolute volume is measured in cm X cm X cm X 0.54 (spheric T vol), or 0.785 for cylindric T vol)

d) Tumour location was defined in accordance with the definition used in the Smeland *et al.* (2019) analysis of survival and prognosis in the EURAMOS-1 trial. Information was pooled from study variables 'site' (eg. femur, pelvis, spine, etc.) and 'location' (e.g. proximal, axial, etc.). Observed axial tumour locations included rib (14) and pelvis/sacrum (41).

Statistical analysis

Multi-state model

In the EURAMOS data, information on various post-surgical events was recorded, with up to two intermediate events reported per patient. Supplementary Fig. 1 (Appendix A) gives an overview of all events and transitions recorded in the data. We selected all intermediate states and transitions that had sufficient events to ensure reliable estimation. We distinguish the following intermediate events: local recurrence (LR), new metastatic disease (NM), the combination of both (LR+NM) and secondary malignancy (SM). New metastatic disease (NM) includes new pulmonary metastases (60%), bone metastases (7%), metastases at other sites (19%) and any combination of the three (14%).

In our model, surgical resection of the primary tumour was chosen as a starting state, with 1631 patients. Of these, 526 experienced an intermediate event, moving to the states of either LR (n=61), NM (n=407), LR+NM (n=35), or SM (n=23), with 280 patients transitioning to death afterwards. An additional 25 patients died without experiencing an intermediate event. Of the 407 patients experiencing a new metastatic disease, 55 experienced a second metastatic disease (NM2) after remission, with 33 patients dying subsequently.

For peer review only - http://bmjopen.bmj.com/site/about/guidelines.xhtml

Figure 2 shows a schematic representation of the fitted multi-state model, which consists of seven states and 10 transitions. Appendix A, with supplementary Fig. 1, 2 and 3, provides additional detail on model choice and the definition of states and transitions.

Time varying effect

The model was stratified on transition, allowing a separate baseline for each of the seven transitions. Histological response violated the proportional hazards assumption for the transitions from surgery to NM and NM to death, indicating that the hazard did not remain constant over time. To account for the time-varying effect, an interaction term of histological response with the exponent of time was included.

Missing data

Of the 1631 patients, 1264 patients were complete cases, with the greatest missingness observed for absolute tumour volume (19%). To make full use of the available data, missing values were imputed in a 10-fold imputation approach. Pooled estimates of the coefficients and standard deviations were obtained using Rubin's rule.[8] For the predictor surgical excision an 'unknown' excision was reported for 16 patients. A sensitivity analysis was conducted by evaluating estimate consistency across three different models, with unknown excision patients excluded from the data, with unknown excision modelled as a separate predictor category, and with unknown excisions treated as missing data and imputed. In the absence of substantial differences in model estimates the latter option was selected.

The statistical analysis was performed in the R-software environment (R version 3.4.2)[9], with the *mstate*[10] and *Amelia*[11] libraries.

Patient and public involvement

Patients or the public were not involved in the design, conduct, reporting or dissemination plans of our research (a re-analysis of the EURAMOS-1 trial using a multi-state approach).

For peer review only - http://bmjopen.bmj.com/site/about/guidelines.xhtml

Results

Transition-specific hazard ratios

The multi-state model is illustrated in Figure 2, along with the number of events. Hazard ratios (HR) with 95% confidence intervals (CI) were estimated with a multivariate Cox proportional hazard regression model, modelling all transitions with sufficient events (Supplementary Table 1, Appendix A). Table 2 and 3 show the estimates for all transitions from surgery to intermediate event, and for all transitions terminating in death, respectively.

An axial tumour site, compared to any other limb site except proximal, was associated with a highly increased risk of LR after surgery (HR=10.84, 95% CI: 8.46-13.86, Table 2), death after LR (HR=11.54, 95% CI: 6.11 -21.78, Table 3), and event-free death, i.e. patients who move directly from surgery to death (HR=6.92, 95% CI: 4.50-10.65, Table 3), and with a moderate increased risk of death after NM (HR=1.34, 95% CI: 1.02-1.76, Table 3). Patients with an axial tumour were comparatively less likely to experience an NM after surgery (HR=0.70, 95% CI: 0.56-0.87, Table 2).

Proximal tumour location was associated with moderately increased risks of LR (HR=1.60, 95% CI: 1.21-2.12), NM (HR=1.41, 95% CI: 1.28-1.56), event-free death (HR=2.30, 95% CI: 1.59-3.34), death after LR (HR=2.72, 95% CI: 1.70-4.35), and death after NM (HR=1.33, 95% CI: 1.15-1.53) .

A poor histological response was associated with an increased risk of NM (HR=5.81, 95% CI: 5.31-6.36), LR+NM (HR=3.82, 95% CI: 2.86-5.10), SM (HR=2.07, 95% CI: 1.48-2.90) and LR (HR=1.76, 95% CI: 1.44-2.13). Poor histological response was also associated with an increased risk of event-free death (HR=1.55, 95% CI: 1.15-2.10), and an increased risk of death in patients who had experienced LR+NM (HR=2.45, 95% CI: 1.56-3.86), only LR (HR=1.75, 95% CI: 1.24-2.47), or NM (HR=1.47, 95% CI: 1.29-1.70).

An intralesional surgical excision, compared to a wide or radical one, was strongly associated with an increased risk of a second NM (HR=4.10, 95% CI: 2.61-6.43), death after experiencing a second NM (HR=5.30, 95% CI: 2.86-9.29), and more modestly with LR after surgery (HR=2.08, 95% CI: 1.32-3.29). Patients with either an intralesional or marginal surgical excision were less likely to progress from LR to death (HR=0.33, 95% CI: 0.16-0.67; HR=0.38, 95% CI: 0.22-0.75, respectively).

Younger patients aged <12, compared to adolescents aged 12-18, had a decreased risk of LR (HR=0.66, 95% CI: 0.51-0.86), NM (HR=0.80, 95% CI: 0.72-0.88), SM (HR=0.61, 95% CI: 0.42-0.89), and event-free

For peer review only - http://bmjopen.bmj.com/site/about/guidelines.xhtml

Table 2. Hazard ratio estimates from multi-state model per transition, for all transitions from surgery to intermediate event

	Surgery ➔ Local recurrence		Surgery ➔ New metastatic disease		Surgery ➔ New metastatic disease + Local recurrence		Surgery ➔ Secondary malignancy		New metastatic disease ➔ New metastatic disease 2	
Predictor	**HR**	**95% CI**	**HR**	**95% CI**	**HR**	**95% CI**	**HR**	**95% CI**	**HR**	**95% CI**
Age										
12-18	1		1		1		1		1	
<12	**0.662**	**0.509 - 0.861**	**0.798**	**0.724 - 0.88**	0.844	0.612 - 1.165	**0.612**	**0.42 - 0.893**	1.024	0.793 - 1.323
>18	0.868	0.688 - 1.094	1.088	0.996 - 1.187	0.933	0.689 - 1.265	**0.575**	**0.363 - 0.91**	**0.672**	**0.519 - 0.869**
Histological response										
Good (<10% tumour)	1		1		1		1		1	
Poor (≥10% tumour)	**1.755**	**1.444 - 2.133**	**5.814**	**5.314 - 6.361**	**3.818**	**2.856 - 5.104**	**2.072**	**1.479 - 2.904**	1.052	0.859 - 1.289
Time effect			**0.986**	**0.985 - 0.987**						
Excision										
Wide/radical	1		1						1	
Marginal	0.779	0.587 - 1.033	1.096	0.985 - 1.219					0.862	0.625 - 1.189
Intralesional	**2.082**	**1.32 - 3.285**	1.212	0.938 - 1.566					**4.095**	**2.609 - 6.428**
Location										

death (HR=0.56, 95% CI: 0.37-0.84). However, younger patients had an increased risk of death after LR (HR=2.40, 95% CI: 1.52-3.90), LR+NM (HR=2.84, 95% CI: 1.83-4.41), and NM2 (HR=2.34, 95% CI: 1.69-3.22). Patients older than 18 years experienced a decreased risk of SM (HR=0.58, 95% CI: 0.36-0.91), NM2 (HR=0.67, 95% CI: 0.52-0.87), death after LR (HR=0.35, 95% CI: 0.21-0.56), death after LR+NM (HR=0.47, 95% CI: 0.31-0.71), and death after NM2 (HR=0.53, 95% CI: 0.34-0.83).

Male sex was associated with an increased risk of LR+NM (HR=1.40, 95% CI: 1.08-1.82), NM2 (HR=1.34, 95% CI: 1.09-1.65), death after NM (HR=1.59, 95% CI: 1.42-1.79), and death after LR+NM (HR=1.82, 95% CI: 1.30-2.55), but a decreased risk of SM (HR=0.72, 95% CI: 0.53-0.98).

A tumour volume of >200 was associated with a modest increase in risk of NM (HR=1.51, 95% CI: 1.40-1.64) and LR+NM (HR=1.50, 95% CI: 1.15-1.95), and a decrease in risk of NM2 (HR=0.76, 95% CI: 0.59-0.98).

For peer review only - http://bmjopen.bmj.com/site/about/guidelines.xhtml

Other	1		1							
Proximal femur/humerus	**1.601**	**1.208 - 2.123**	**1.410**	**1.278 - 1.556**						
Axial	**10.837**	**8.458 - 13.885**	**0.700**	**0.564 - 0.869**						
Sex										
Female	1		1		1		1		1	
Male	0.947	0.783 - 1.145	1.048	0.972 - 1.13	**1.402**	**1.079 - 1.82**	**0.724**	**0.534 - 0.982**	**1.343**	**1.092 - 1.652**
Volume										
<200	1		1		1		1		1	
≥200	0.802	0.642 - 1.001	**1.513**	**1.397 - 1.638**	**1.499**	**1.152 - 1.951**	0.755	0.502 - 1.134	**0.760**	**0.592 - 0.975**

Table 3. Hazard ratio estimates from multi-state model per transition, for all absorbent transitions from intermediate event to death

	Surgery ➔ Death		Local recurrence ➔ Death		New metastatic disease ➔ Death		New metastatic disease + Local recurrence ➔death		New metastatic disease 2 ➔ Death	
Predictor	**HR**	**95% CI**	**HR**	**95% CI**	**HR**	**95% CI**	**HR**	**95% CI**	**HR**	**95% CI**
Age										
12-18	1		1		1		1		1	
<12	**0.555**	**0.368 - 0.836**	**2.401**	**1.517 - 3.799**	0.881	0.757 - 1.024	**2.838**	**1.825 - 4.413**	**2.335**	**1.694 - 3.218**
>18	0.739	0.506 - 1.081	**0.345**	**0.213 - 0.560**	0.892	0.785 - 1.014	**0.473**	**0.314 - 0.711**	**0.529**	**0.338 - 0.829**
Histological response										
Good (<10% tumour)	1		1		1		1		1	
Poor (≥10% tumour)	**1.551**	**1.147 - 2.099**	**1.748**	**1.236 - 2.471**	**1.472**	**1.29 - 1.679**	**2.453**	**1.561 - 3.855**	0.872	0.645 - 1.179
Time effect					**0.979**	**0.967 - 0.992**				
Excision										
Wide/radical			1		1				1	
Marginal			**0.379**	**0.221 - 0.651**	1.089	0.929 - 1.277			0.811	0.442 - 1.490
Intralesional			**0.331**	**0.163 - 0.673**	1.401	0.975 - 2.014			**5.296**	**2.856 - 9.819**
Location										

For peer review only

For peer review only - http://bmjopen.bmj.com/site/about/guidelines.xhtml

Other	1		1		1					
Proximal femur/humerus	**2.299**	**1.585 - 3.335**	**2.717**	**1.696 - 4.354**	**1.326**	**1.151 - 1.527**				
Axial	**6.919**	**4.497 - 10.65**	**11.535**	**6.113 - 21.767**	**1.335**	**1.016 - 1.755**				
Sex										
Female	1		1		1		1		1	
Male	0.986	0.733 - 1.326	1.305	0.923 - 1.844	**1.589**	**1.418 - 1.782**	**1.823**	**1.302 - 2.552**	1.165	0.836 - 1.623
Volume										
<200	1		1		1		1		1	
≥200	1.093	0.772 - 1.548	0.756	0.516 - 1.106	1.03	0.915 - 1.16	1.13	0.804 - 1.589	0.866	0.591 - 1.271

For peer review only

Time-varying effect

Histological response violated the proportional hazards assumption for the transition surgery to NM and NM to death. Therefore, the effect of histological response is modelled as function of time. The HR of transitioning to NM decreased with time, initially slowly, and more quickly around the three-year mark (Figure 3A). For the transition from NM to death, the decrease was less pronounced (Figure 3B). The time-varying hazard ratio can be computed for any given time-point using the formula shown below. For example, the HR for the transition from surgery to NM is given as:

$$\text{HR} = \text{constant} \times \text{time-varying-effect}^{\exp(t)}.$$

Here, *constant* is the surgery to NM HR of 5.81, the *time-varying effect* is 0.986 (Table 2), and *t* is time in years. At 1 year, the hazard ratio is: HR= $5.81 \times 0.985^{\exp(1)} = 5.76$. At three years, the HR has decreased to $5.81 \times 0.985^{\exp(3)} = 4.36$.

State-occupation probability plots

Figure 4 illustrates, for a set of example patients, osteosarcoma disease progression through state occupation probability plots, where the probability of being in a given state is plotted against time, in years since surgery. Whereas hazard ratios only inform on the relative change in risk, state occupation probabilities show the absolute probability of being in a given state (i.e., having experienced an

intermediate event) over a period of time. Panels 1A to 1C illustrate the effect of age category, with in panel 1A the state occupation probabilities of a patient aged 12-18, with all other characteristics reference categories (good histological response, wide/radical excision, tumour volume <200 cm^3, female, tumour location of category 'other'), and in panels 1B and 1C the state occupation probabilities of patients aged <12 and >=18, respectively. Patients aged 12-18 were least likely to remain event-free in the state of surgery (green) and had a moderately higher probability of death (purple). Patients aged <12 were most likely to remain event-free with a lower probability of an intermediate event or death. Patients aged >=18 were at greater risk of NM (blue) than both other age groups.

When compared to the reference patient (1A), patients with an axial osteosarcoma (2A) were at lower risk of NM, higher risk of death, and a much-increased risk of LR (yellow), and were most likely to experience the latter event 1 to 3 years after surgery. An intralesional surgical excision (2B), in contrast, resulted an in increased risk of LR that remained relatively constant over time. Patients with a poor histological response (2C) were more likely to experience NM, or an SM (red). Panels 3A-3C and 4A-4C show the state occupation probabilities for patients who experienced an NM or LR, respectively, at 1, 2 and 4 years after surgery. The later the event occurred, the lower the probability of the disease progressing to death. For example, for LRs 1 year and 4 years after surgery, the 4-year survival probabilities were approximately 0.65 (4A) and 0.8 (4C), respectively.

Discussion

This study is unique in considering the hazard of transitions to and from different intermediate events, allowing for a better insight into prognosis of specific patient groups compared to overall prognostic factors. Using a metastasis-free subset of the EURAMOS-1 data[6], we interpret the effect of six prognostic factors on osteosarcoma disease progression after surgery in a multi-staged way. Multi-state model estimation is in practice limited to transitions with a sufficient number of events per predictor category. Predictor categories with few patients (e.g., axial tumour location and intralesional surgical excision), when combined with rare intermediate events (e.g., NM+LR and SM), will preclude statistical inference. Specifically, we were unable to estimate the effect of prognostic factors surgical excision and tumour location for four and five out of a total of 10 transitions, respectively.

For peer review only - http://bmjopen.bmj.com/site/about/guidelines.xhtml

The most remarkable adverse risk factor was axial location of the osteosarcoma. A previous analysis of the EURAMOS-1 data found moderate HRs of 1.74 (95% CI: 1.06-2.85) and 1.29 (95% CI: 0.86-1.95) for overall survival (OS) and event-free survival (EFS), respectively.[6] In our results, an axial location was chiefly relevant to the transition from surgery to LR and LR to death, yielding substantially higher transition-specific hazard ratios of 10.84 (95% CI: 8.45-13.86) and 11.54 (95% CI: 6.11-21.77), respectively. In contrast, the occurrence of an NM was less common (HR=0.70, 95% CI: 0.56-0.87). Clinically, this may have implications for additional treatment and follow-up for osteosarcomas at this site.

A poor histological response has widely been shown to adversely affect overall survival. [6,12-14] The previous EURAMOS-1 analysis found HRs of 2.14 (95% CI: 1.76-2.58) and 2.45 (95% CI: 1.88-3.20) for EFS and OS, respectively. In our study, poor histological response increased the risk of events NM nearly 6x (HR 5.81, 95% CI: 5.31-6.36) and LR+NM nearly 4x (HR 3.81, 95% CI: 2.86-5.10), was predictive of death chiefly after an immediate event, but only modestly affected the risk of an event-free death. In particular, the risk of NM was substantially increased during the first 2.5 years, but the predictive strength steeply decreased after 3 years since surgery, with the HR nearly halved at 4 years follow-up.

For an intralesional excision, compared to a wide/radical excision, HRs of 2.73 (95% CI: 1.15-6.47) and 1.98 (95% CI: 0.91-4.30) were previously found for OS and EFS, respectively.[6] Our results confirm a higher probability of an LR after intralesional surgery (HR 2.08, 95% CI: 1.32-3.29), with this risk remaining comparatively constant during follow-up. Our study shows that developing recurrent NM is a risk factor of intralesional surgery (HR 4.10, 95% CI: 2.61-6.43), with the risk of a subsequent poor outcome increased more than 5x (HR 5.30, 95% CI: 2.86-9.82), substantially higher than the previously reported OS HR.

The remaining variables in our model had more modest HRs, which varied less across transitions. Previously, male sex was found to have only a modest adverse effect on EFS and OS.[6,15] We observed an increased risk of LR+NM and NM2, and subsequent death, but a decreased risk of SM. For greater tumour volume, an EFS HR of 1.24 (95% CI: 1.00-1.52) was previously found[6]. We observed an increased risk for the events NM and LR+NM, with no significant effect for any transition to death, which is in line with previous results for OS (HR=1.19, 95% CI: 0.92-1.55).[5]

For peer review only - http://bmjopen.bmj.com/site/about/guidelines.xhtml

Young age was found to be protective for OS and EFS in the previous EURAMOS-1 analysis[6], and other studies.[15,16] However, in Collins' study[15], the hazard lost its significance for survival, at 2 years post-surgery, and our study did not confirm increased risk of event. We found that both the youngest patients (< 12 yr) and those > 18 were protected from experiencing an event. Given the occurrence of LR, LR+NM or NM2, the probability of poor outcome was highest in the young age group, whereas patients > 18 year had a remarkably better outcome. This may have implications for the treatment of LR in younger versus older patients, suggesting that older patients could be treated by local treatment only, whereas for younger patients, a more intensified treatment regimen with additional systemic treatment should be considered.

Conclusion

Using data of more than 1600 patients from the EURAMOS-1 trial, we estimated a multi-state model with intermediate events. This study shows the added value of considering prognostic factors specific to transition and in light of event history. Our findings indicate that young patients with a local recurrence have a poor prognosis, suggesting that it may be beneficial to investigate additional treatment options for this subgroup. Additionally, our results stress the necessity of increased monitoring of patients with axial tumours for local recurrences, and patients with a poor histological response for new metastatic disease, while noting for the latter that predictive power decreases over time. We show that a multi-state model yields additional clinical knowledge for specific osteosarcoma patient groups, when compared to conventional OS and EFS analyses. Previously, multi-state models have been used to model disease progression in soft tissue sarcoma[17,18], Ewing sarcoma[19] and breast cancer[20]. Re-analyzing data from other large randomised studies, using the multi-state approach, may also yield valuable insights for patients with other oncological conditions.

Contributorship statement

Audinga-Dea Hazewinkel: Writing- original draft preparation, Visualization, Formal analysis, Software. *Carlo Lancia:* Data curation, Writing- Review & Editing. *Jakob Anninga*: Writing- Review & Editing. *Michiel van de Sande*: Writing- Review & Editing. *Jeremy Whelan*: Investigation. *Hans Gelderblom*:

For peer review only - http://bmjopen.bmj.com/site/about/guidelines.xhtml

Conceptualization, Writing- Review & Editing. *Marta Fiocco:* Conceptualization, Supervision, Writing-Review & Editing

Competing interests

The authors declare no conflict of interest.

Funding

We thank the EURAMOS-1 trial investigators and participants.

This work was supported by the Dutch Foundation KiKa (Stichting Kinderen Kankervrij), grant 163, through the project *Meta-analysis of individual patient data to investigate dose-intensity relation with survival outcome for osteosarcoma patients*. AH was supported by Integrative Epidemiology Unit, which receives funding from the UK Medical Research Council and the University of Bristol (MC_UU_00011/3).

Data sharing statement

A request to access the EURAMOS-1 trial data may be submitted to the MRC Clinical Trials Unit (CTU, London). The application requires completion of an *analysis and data release request form*, where the applicant provides a project summary (detailing the motivation of the data request, the background and objectives of their project, and the reasons for requesting this specific dataset), the data requirements (for this study, the anonymized individual level data for all registered patients were requested, including (demographic) patient characteristics, disease characteristics, pathology and surgical information, treatment data, and major events), and details on the proposed publication, authorship and acknowledgements policy. Data applications are submitted to the Coordinating Data Center (CDC, London), and subject to review by the Trial Management Group (TMG) and the Trial Steering Committee (TSC).

Ethics statement

Permission to recruit patients to the protocol was provided by the appropriate national and local regulatory and local committees. Link anonymized data were used for the purposes of this study, and the use of the data was in accordance with the consent taken.

For peer review only - http://bmjopen.bmj.com/site/about/guidelines.xhtml

References

1. Gorlick R, Bielack S, Teot L, et al: Osteosarcoma: Biology, diagnosis, treatment, and remaining challenges, in Pizzo P, Poplack D (eds): *Principles and Practice of Pediatric Oncology* 6: 1015-1044, 2011
2. Isakoff MS, Bielack SS, Meltzer P, et al. Osteosarcoma: Current Treatment and a Collaborative Pathway to Success. *J Clin Oncol* 33(27):3029-3035, 2015
3. Whelan JS, Bielack SS, Marina N, et al: EURAMOS-1, an international randomised study for osteosarcoma: Results from pre-randomisation treatment. *Ann Oncol* 26:407-414, 2015
4. Bielack SS, Smeland S, Whelan JS, et al: Methotrexate, Doxorubicin, and Cisplatin (MAP) Plus Maintenance Pegylated Interferon Alfa-2b Versus MAP Alone in Patients With Resectable High-Grade Osteosarcoma and Good Histologic Response to Preoperative MAP: First Results of the EURAMOS-1 Good Response Randomized Controlled Trial. *Journal of Clinical Oncology* 33: 2279–2287, 2015
5. Marina, NM, Smeland S, Bielack SS, et al: Comparison of MAPIE versus MAP in patients with a poor response to preoperative chemotherapy for newly diagnosed high-grade osteosarcoma (EURAMOS-1): an open-label, international, randomised controlled trial. *The Lancet, Oncology* 17: 1396–1408, 2016
6. Smeland S, Bielack SS, Whelan J, et al. Survival and prognosis with osteosarcoma: outcomes in more than 2000 patients in the EURAMOS-1 (European and American Osteosarcoma Study) cohort. *Eur J Cancer*. 2019;109:36-50.
7. Putter H, Fiocco M, R Geskus. Tutorial in biostatistics: competing risks and multi-state models. Stat Med 26:2389–430, 2007
8. Rubin, DB: Multiple imputation after 18+ years. *Journal of the American statistical association.* 91(434):473-489, 1996.
9. R: A language and environment for statistical computing. R Foundation for Statistical Computing, Vienna, Austria. http://www.R-project.org
10. Liesbeth C. de Wreede, Marta Fiocco, Hein Putter. Mstate: An R Package for the Analysis of Competing Risks and multi-state models. *Journal of Statistical Software.* 38(7):1-30, 2011
11. Honaker J, King G, Blackwell M. "Amelia II: A Program for Missing Data." *Journal of Statistical Software*. 45(7):1–47, 2011
12. Rosen G, Murphy ML, Huvos AG, et al: Chemotherapy, en bloc resection, and prosthetic bone replacement in the treatment of osteogenic sarcoma. *Cancer.* 37:1–11, 1976
13. Bacci G, Picci P, Ruggieri P, et al: Primary chemotherapy and delayed surgery (neoadjuvant chemotherapy) for osteosarcoma of the extremities. *Cancer*. 65:2539–2553, 1990
14. Whelan JS, Jinks RC, McTiernan A, et al: Survival from high-grade localised extremity osteosarcoma: combined results and prognostic factors from three European Osteosarcoma Intergroup randomised controlled trials. *Ann Oncol* 23:1607–1616, 2012
15. Collins M, Wilhelm M, Conyers R, et al: Benefits and adverse events in younger versus older patients receiving neoadjuvant chemotherapy for osteosarcoma: findings from a meta-analysis. *J Clin Oncol* 31:2303-12, 2013.
16. Hagleitner MM, Hoogerbrugge PM, van der Graaf WT, et al: Age as prognostic factor in patients with osteosarcoma. *Bone* 49:1173-7, 2012
17. Willeumier JJ, Rueten-Budde AJ, Jeys LM, et al: Individualised risk assessment for local recurrence and distant metastases in a retrospective transatlantic cohort of 687 patients with high-grade soft tissue sarcomas of the extremities: a multistate model. *BMJ Open*. 2017;7:e012930.
18. Posch F, Leitner L, Bergovec M, et al. Can Multistate Modeling of Local Recurrence, Distant Metastasis, and Death Improve the Prediction of Outcome in Patients With Soft Tissue Sarcomas?. *Clin Orthop Relat Res*. 2017;475(5):1427-1435. doi:10.1007/s11999-017-5232-x

For peer review only - http://bmjopen.bmj.com/site/about/guidelines.xhtml

19. Bosma SE, Rueten-Budde AJ, Lancia C, et al. Individual risk evaluation for local recurrence and distant metastasis in Ewing sarcoma: A multistate model: A multistate model for Ewing sarcoma. *Pediatr Blood Cancer*. 2019;66(11):e27943. doi:10.1002/pbc.27943
20. Putter H, van der Hage J, de Bock GH, Elgalta R, van de Velde CJ. Estimation and prediction in a multi-state model for breast cancer. *Biom J*. 2006;48(3):366-380. doi:10.1002/bimj.200510218

Figure legends

For peer review only

Figure 1 Consort diagram of patients included in the analysis. In the boxes we list the six exclusion criteria with the total number of patients per category. Above the arrows, we give the additional number of patients excluded on considering each criterion, in order of appearance. a) to ensure a homogeneous study population, we excluded patients with metastases prior to surgery. For 357 patients, metastases were recorded at registration, while for 170 patients, progression of new metastatic disease was found after surgery, while no metastases were recorded at registration. These patients were retrospectively reclassified as having metastatic disease prior to surgery and excluded from the analysis; b) 22 randomized patients were later found to be ineligible due to progression of metastatic disease or new metastatic disease (n=11), or primary and/or metastatic unresectable disease (n=11).

Figure 2 Disease progression of osteosarcoma represented in a multi-state model. Seven possible states and 10 transitions are defined. For each transition, the number of patients progressing from one state to another is shown. A total of 1631 patients are present in the starting stage, Surgery. After surgery, a patient may experience a local recurrence (LR), a local recurrence + new metastatic disease (LR+NM), a new metastatic disease (NM), a secondary malignancy (SM) or death. After any such intermediate event, a patient may progress to death. NM patients may experience a second new metastatic disease after remission (NM2).

Figure 3 Time-varying hazard for histological response. *A*: hazard for transitioning from surgery to new metastatic disease (NM). *B*: hazard for transitioning from NM to death. *Blue*: poor histological response; *black*: good histological response; *Dashed line*: point-wise confidence interval for hazard ratio of poor histological response.

Figure 4 Stacked state occupation probabilities for patients with different characteristics. Patient characteristics are defined with respect to the reference patient, shown in *1A*: patient with reference characteristics – age 12-18

For peer review only - http://bmjopen.bmj.com/site/about/guidelines.xhtml

years, good histological response, wide/radical excision, tumour volume <200 cm^3, female, tumour location of category 'other'. *1B*: patient aged <12 years. *1C*: patient aged >=18 years. *2A:* Patient with axial tumour location. *2B*: Patient with poor histological response. *2C*: patient with intralesional excision. *3A-3C*: Patient with reference characteristics experiencing a new metastatic disease (NM) at one year *(3A)*, at two years (*3B)*, and at four years (*3C*). *4A-4C*: Patient with reference characteristics experiencing local recurrence (LR) at one year *(3A)*, at two years (*3B)*, and at four years (*3C*).

For peer review only

For peer review only - http://bmjopen.bmj.com/site/about/guidelines.xhtml

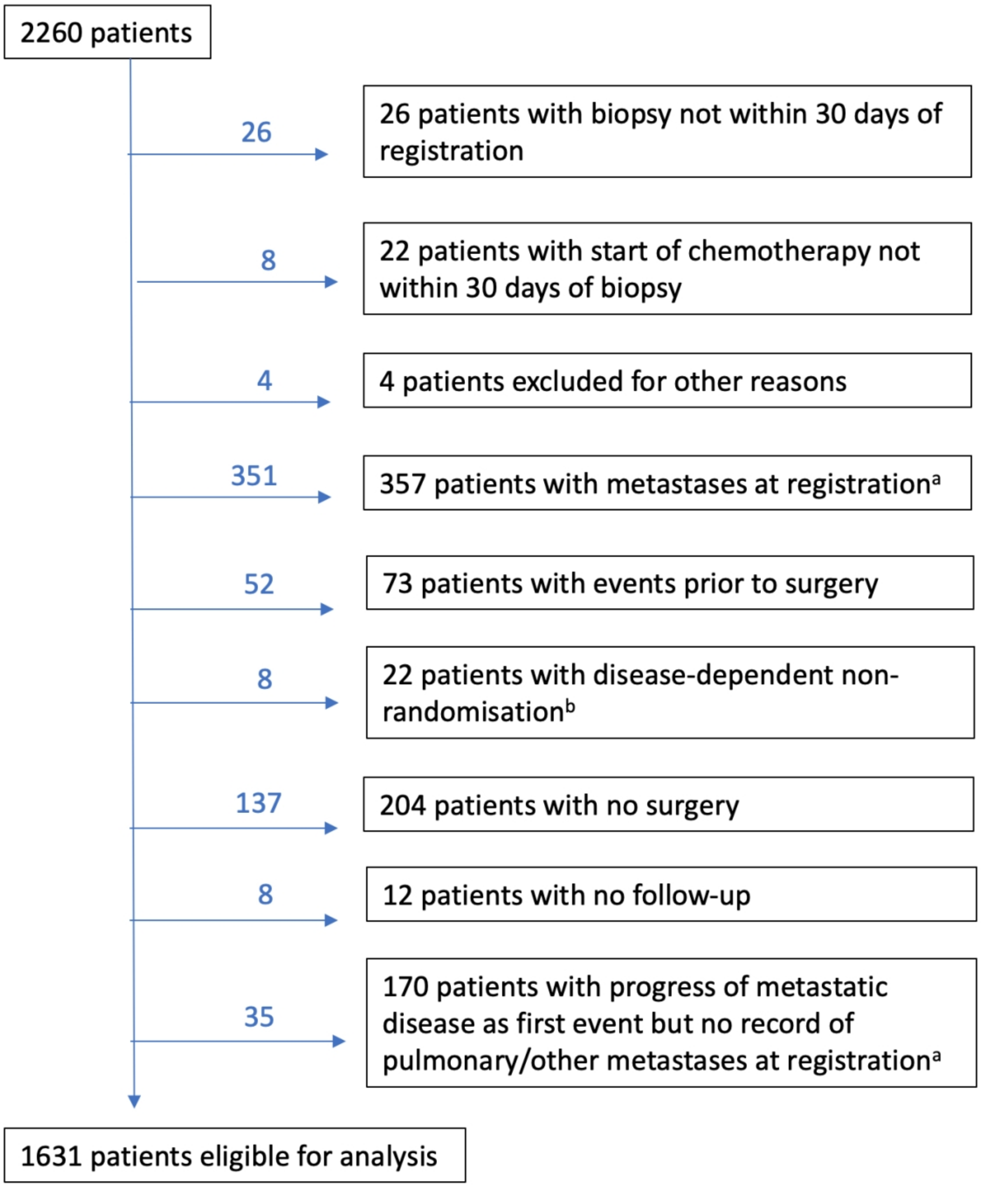

Figure 1 Consort diagram of patients included in the analysis. In the boxes we list the six exclusion criteria with the total number of patients per category. Above the arrows, we give the additional number of patients excluded on considering each criterion, in order of appearance. a) to ensure a homogeneous study population, we excluded patients with metastases prior to surgery. For 357 patients, metastases were recorded at registration, while for 170 patients, progression of new metastatic disease was found after surgery, while no metastases were recorded at registration. These patients were retrospectively reclassified as having metastatic disease prior to surgery and excluded from the analysis; b) 22 randomized patients were later found to be ineligible due to progression of metastatic disease or new metastatic disease (n=11), or primary and/or metastatic unresectable disease (n=11).

239x284mm (144 x 144 DPI)

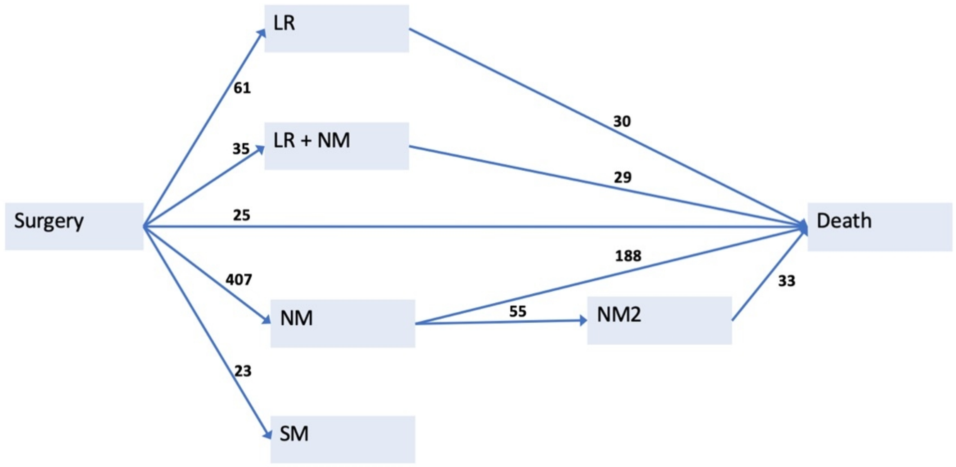

Figure 2 Disease progression of osteosarcoma represented in a multi-state model. Seven possible states and 10 transitions are defined. For each transition, the number of patients progressing from one state to another is shown. A total of 1631 patients are present in the starting stage, Surgery. After surgery, a patient may experience a local recurrence (LR), a local recurrence + new metastatic disease (LR+NM), a new metastatic disease (NM), a secondary malignancy (SM) or death. After any such intermediate event, a patient may progress to death. NM patients may experience a second new metastatic disease after remission (NM2).

141x72mm (220 x 220 DPI)

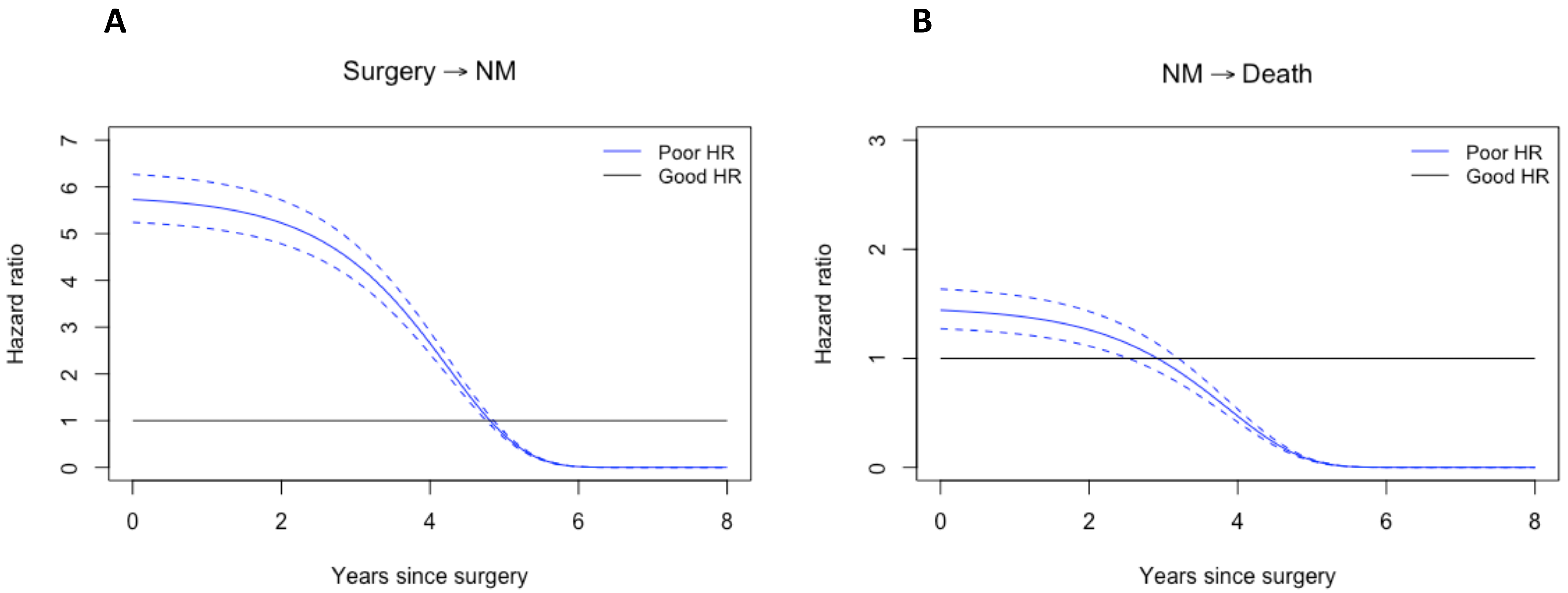
A
Surgery → NM
Hazard ratio
Years since surgery
Poor HR
Good HR
0
1
2
3
4
5
6
7
0
2
4
6
8
B
NM → Death
Hazard ratio
Years since surgery
Poor HR
Good HR
0
1
2
3
0
2
4
6
8

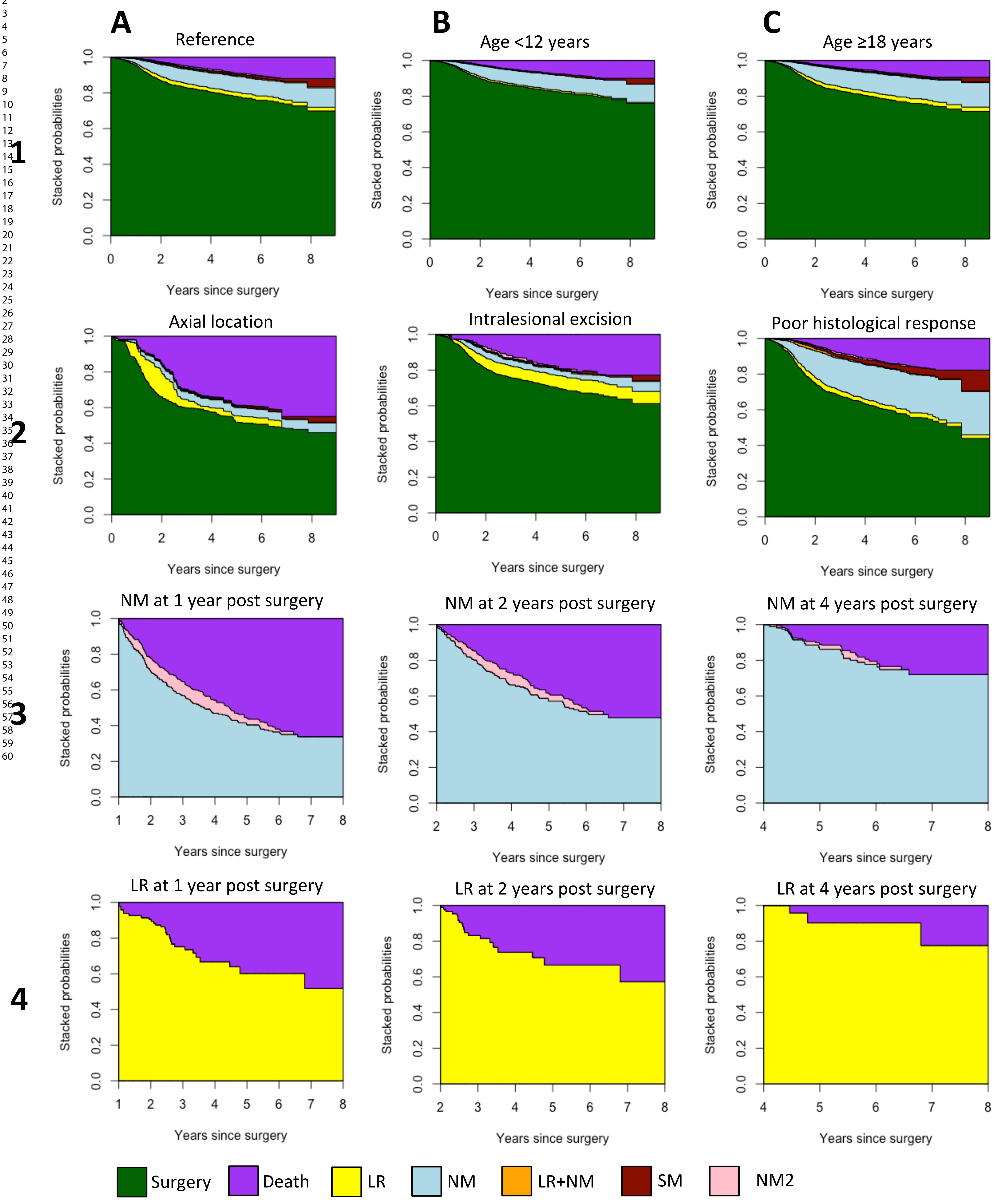

A
B
C
1
2
3
4
Reference
Age <12 years
Age ≥18 years
Axial location
Intralesional excision
Poor histological response
NM at 1 year post surgery
NM at 2 years post surgery
NM at 4 years post surgery
LR at 1 year post surgery
LR at 2 years post surgery
LR at 4 years post surgery
Stacked probabilities
Years since surgery
Surgery
Death
LR
NM
LR+NM
SM
NM2

Appendix A: Defining states and transitions for the EURAMOS-1 osteosarcoma multi-state model

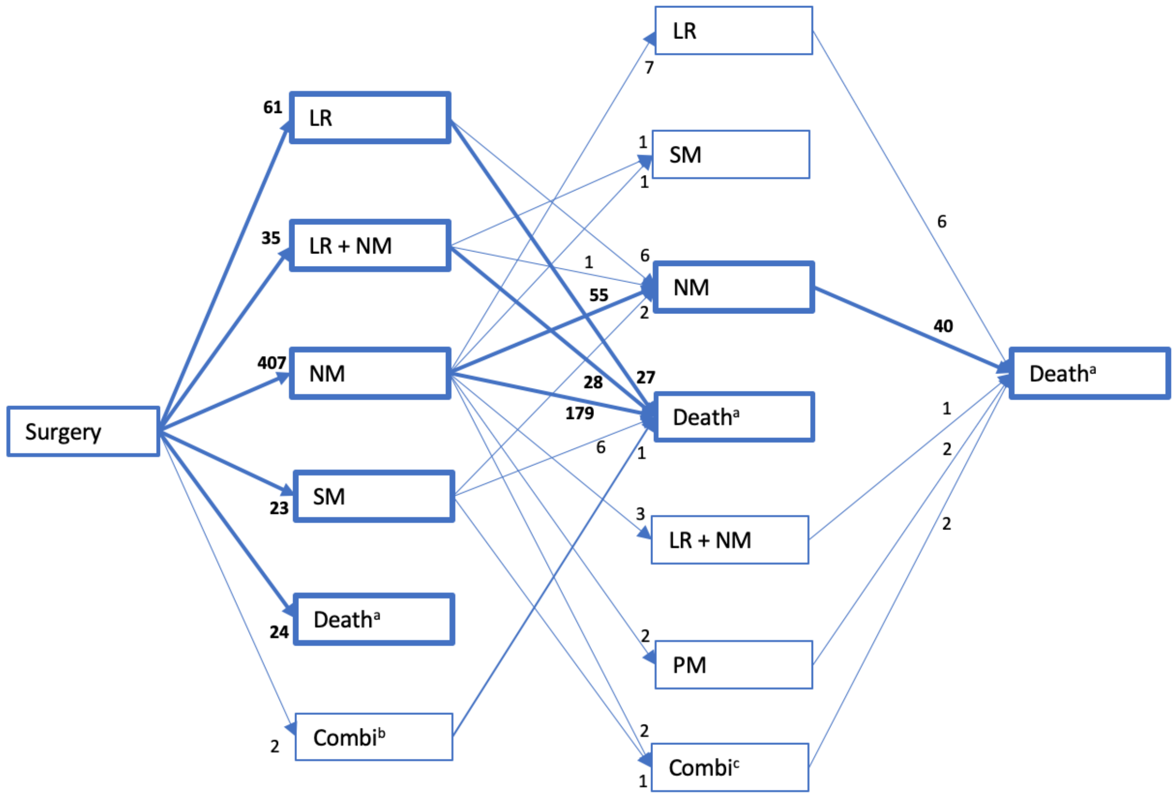

Supplementary Fig. 1 Disease progression of osteosarcoma: all recorded transitions. Eleven possible states and 27 transitions are defined. For each transition, the number of patients progressing from one state to another is shown. A total of 1631 patients are present in the starting stage, Surgery. a) Death is defined as death due to osteosarcoma. Death takes precedence over any other events registered at the same time (e.g. a patient with the combination of NM + Death is considered part of the transition from Surgery to Death, but not the transition from Surgery to NM); b) Combination event: 3 patients with NM+PM; c) Combination event: 1 patient with LR+SM, 1 patient with NM+SM.

Supplementary Fig. 1 gives an overview of all events and transitions recorded in the EURAMOS-1 osteosarcoma data. All patients were considered from their time of surgery, after which a maximum of two consecutive intermediate events was recorded. Our dataset includes a total of 1631 patients who had their primary tumour surgically resected. Of these patients, 552 experienced at least one event, which may be local recurrence (LR, 61), a new metastatic disease (NM, 407), the combination of a local recurrence and a new metastatic disease (LR+NM, 35), a secondary malignancy (SM, 23), a combination of any of the previous events recorded at the same time point (Combi, 2), or death. For example, 24 patients died after surgery without experiencing any of the previously listed events. Another 63 patients experienced a local recurrence, of which 27 died (i.e. transitioned to the state of death), while 6 experienced an NM, and a single patient experienced a second LR.

For peer review only - http://bmjopen.bmj.com/site/about/guidelines.xhtml

In our multi-state model, we have only included transitions with sufficient events. The relevant transitions and corresponding states have been marked in bold. Fig. 2 in the main text is a simplified version of the diagram in Supplementary Fig. 1, showing only the states and transitions that are included in our multi-state model. Particular attention must be paid to the definition of these transitions. Consider, for example, the transition from surgery to death, for which we have 25 patients. This particular transition is defined as death without experiencing any of the intermediate events (NM, LR, LR+NM, SM) that are explicitly included in the model. This is not the same as the transition from surgery to death, as defined in Supplementary Fig. 1, where we have 24 patients who die without experiencing any of the recorded intermediate events. For clarity, we have included a diagram of all patients included in the transition from surgery to death, as defined in our multistate model. In Supplementary Fig. 2 we see that there are 24 patients who transition directly from surgery to death, with two patients experiencing a combination event, one of which transitions to death. As the combination event is not included as a state in our model, this patient is counted amongst the surgery-death transition patients, bringing the total to 25 patients.

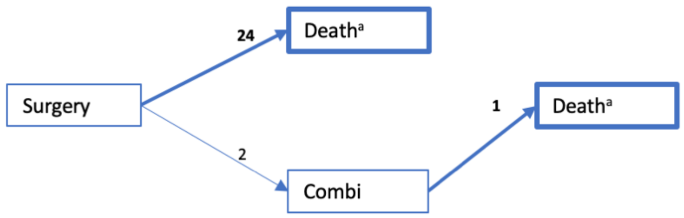

Supplementary Fig. 2 Disease progression of osteosarcoma: the transition from surgery to death, as defined in the multi-state model (Figure 2 in the main text). A total of 25 patients make up the transition from surgery to death in the fitted multi-state model. Of these, 24 progressed to death directly, without any intermediate event, whereas 1 experienced a combination event (NM+PM, not included as a state in the model) prior to death. a) Death is defined as death due to osteosarcoma, and takes precedence over any other events registered at the same time.

Equivalently, we can consider the transition from LR to death, for which we have 30 patients in our multi-state model. In Supplementary Fig. 3 we see that this number is composed of 27 patients who transition directly from LR to death, and 3 patients who experience an NM prior to death. As the latter transition (from LR to NM) is not included in the model, these 3 patients are included in the group of LR-Death transition patients.

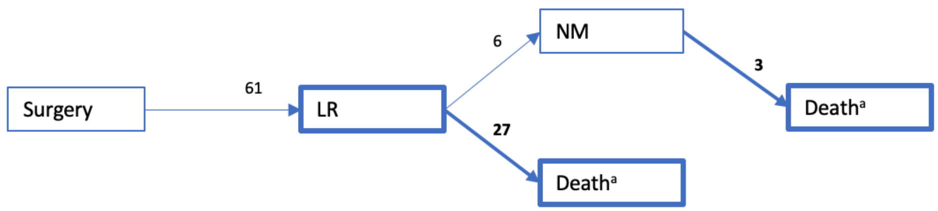

Supplementary Fig. 3 Disease progression of osteosarcoma: the transition from LR to death, as defined in the multi-state model (Figure 2 in the main text). A total of 30 patients make the transition from LR to death in the fitted multi-state model. Of these, 27 progressed to death directly, without any intermediate event, whereas 3 experienced a NM after LR – a transition not included in the multi-state model. a) Death is defined as death due to osteosarcoma, and takes precedence over any other events registered at the same time.

Supplementary Table 1. Event counts per predictor category. Shown are the number of patients per predictor category for each transition. Numbers were computed post multiple imputation, with the final counts averaged across the 10 imputed datasets.

Predictor	Surgery ➔ Local recurrence	Surgery ➔ New metastatic disease	Surgery ➔ New metastatic disease + Local recurrence	Surgery ➔ Secondary malignancy	New metastatic disease ➔ New metastatic disease 2	Surgery ➔ Death	Local recurrence ➔ Death	New metastatic disease ➔ Death	New metastatic disease + Local recurrence ➔death	New metastatic disease 2 ➔ Death
	**Event count**									
Age										
12-18	36	228	20	15	33	16	17	111	17	18
<12	**10**	76	**7**	5	11	4	4	30	7	9
>18	15	103	8	3	11	5	9	47	5	6
Histological response										
Good (<10% tumour)	24	162	8	10	22	11	9	66	6	13
Poor (≥10% tumour)	37	245	27	13	33	14	21	122	23	20
Excision										
Wide/radical	49	338	25	19	45	20	23	156	20	27
Marginal	9	61	9	4	7	5	4	28	8	3
Intralesional	3	8	**1**	**0**	3	**0**	3	4	**1**	3
Volume										
<200	41	246	22	19	39	18	18	107	19	20
≥200	20	161	13	4	16	7	12	81	10	13
Sex										
Female	27	161	12	12	21	11	10	63	10	12
Male	34	246	23	11	34	14	20	125	19	21
Location										
Other	38	328	30	20	49	16	13	146	24	28
Proximal	8	67	5	3	6	5	4	34	5	5
Axial	15	12	**0**	**0**	**0**	4	13	8	**0**	**0**

For peer review only

CONSORT 2010 checklist of information to include when reporting a randomised trial*

Section/Topic	Item No	Checklist item	Reported on page No
**Title and abstract**			
	1a	Identification as a randomised trial in the title	1
	1b	Structured summary of trial design, methods, results, and conclusions (for specific guidance see CONSORT for abstracts)	3
**Introduction**			
Background and objectives	2a	Scientific background and explanation of rationale	5
	2b	Specific objectives or hypotheses	5
**Methods**			
Trial design	3a	Description of trial design (such as parallel, factorial) including allocation ratio	5
	3b	Important changes to methods after trial commencement (such as eligibility criteria), with reasons	NA
Participants	4a	Eligibility criteria for participants	6
	4b	Settings and locations where the data were collected	3
Interventions	5	The interventions for each group with sufficient details to allow replication, including how and when they were actually administered	NA
Outcomes	6a	Completely defined pre-specified primary and secondary outcome measures, including how and when they were assessed	3
	6b	Any changes to trial outcomes after the trial commenced, with reasons	NA
Sample size	7a	How sample size was determined	NA
	7b	When applicable, explanation of any interim analyses and stopping guidelines	NA
Randomisation:			
Sequence generation	8a	Method used to generate the random allocation sequence	NA
	8b	Type of randomisation; details of any restriction (such as blocking and block size)	NA
Allocation concealment mechanism	9	Mechanism used to implement the random allocation sequence (such as sequentially numbered containers), describing any steps taken to conceal the sequence until interventions were assigned	NA
Implementation	10	Who generated the random allocation sequence, who enrolled participants, and who assigned participants to interventions	NA
Blinding	11a	If done, who was blinded after assignment to interventions (for example, participants, care providers, those	NA

For peer review only

		assessing outcomes) and how	
	11b	If relevant, description of the similarity of interventions	NA
Statistical methods	12a	Statistical methods used to compare groups for primary and secondary outcomes	7-8
	12b	Methods for additional analyses, such as subgroup analyses and adjusted analyses	7-8
**Results**			
Participant flow (a diagram is strongly recommended)	13a	For each group, the numbers of participants who were randomly assigned, received intended treatment, and were analysed for the primary outcome	5-6
	13b	For each group, losses and exclusions after randomisation, together with reasons	5-6
Recruitment	14a	Dates defining the periods of recruitment and follow-up	5
	14b	Why the trial ended or was stopped	5
Baseline data	15	A table showing baseline demographic and clinical characteristics for each group	6-7
Numbers analysed	16	For each group, number of participants (denominator) included in each analysis and whether the analysis was by original assigned groups	7
Outcomes and estimation	17a	For each primary and secondary outcome, results for each group, and the estimated effect size and its precision (such as 95% confidence interval)	8-13
	17b	For binary outcomes, presentation of both absolute and relative effect sizes is recommended	NA
Ancillary analyses	18	Results of any other analyses performed, including subgroup analyses and adjusted analyses, distinguishing pre-specified from exploratory	NA
Harms	19	All important harms or unintended effects in each group (for specific guidance see CONSORT for harms)	NA
**Discussion**			
Limitations	20	Trial limitations, addressing sources of potential bias, imprecision, and, if relevant, multiplicity of analyses	13-14
Generalisability	21	Generalisability (external validity, applicability) of the trial findings	13-14
Interpretation	22	Interpretation consistent with results, balancing benefits and harms, and considering other relevant evidence	13-14
**Other information**			
Registration	23	Registration number and name of trial registry	3
Protocol	24	Where the full trial protocol can be accessed, if available	5
Funding	25	Sources of funding and other support (such as supply of drugs), role of funders	2

*We strongly recommend reading this statement in conjunction with the CONSORT 2010 Explanation and Elaboration for important clarifications on all the items. If relevant, we also recommend reading CONSORT extensions for cluster randomised trials, non-inferiority and equivalence trials, non-pharmacological treatments, herbal interventions, and pragmatic trials. Additional extensions are forthcoming: for those and for up to date references relevant to this checklist, see www.consort-statement.org.

For peer review only - http://bmjopen.bmj.com/site/about/guidelines.xhtml
